# Process optimization for protoplast preparation from *Grifola frondosa* and development of a high-yield mutant with enhanced mycelial growth and protein production

**DOI:** 10.1186/s12896-026-01197-z

**Published:** 2026-07-08

**Authors:** Qiaoyu Wang, Ziteng Zhao, Qiuyu Lu, Ke’er Xiao, Lingyu Gao, Mukaddas Sai, Lili Guan, Linna Du

**Affiliations:** Engineering Research Center of Bioreactor and Pharmaceutical Development, Ministry of Education, College of Life Science, Jilin Agricultural University, No. 2888, Xincheng Street, Changchun, 130118 China

**Keywords:** *Grifola frondosa*, Mutagenesis, Production, Protoplast, Optimization

## Abstract

*Grifola frondosa* is a highly valued medicinal mushroom with diverse bioactive properties; however, its low mycelial biomass remains a major bottleneck affecting the efficiency of liquid fermentation. To address this issue, protoplast-mediated mutagenesis was employed in this study to breed a high-performance *G. frondosa* mutant with superior fermentation traits. Following optimization using intelligent strategies, the highest yield of protoplasts was achieved (6.573 × 10^6^ protoplasts/g fresh weight) under suitable isolation parameters (fungal age of 7.9 days, lywallzyme concentration 2.1%, enzymatic hydrolysis time 5.2 hours, enzymatic temperature of 30 °C, and mannitol concentration of 0.6 mol/L). Subsequently, DES-induced protoplast mutagenesis yielded a stable *G. frondosa* mutant (D24) showing 62.78% greater mycelial biomass and 111.11% higher protein yield relative to the parental strain. Furthermore, fermentation kinetics profiling and phenotypic observation suggested that the mutant D24 exhibited a shortened lag phase, enhanced hyphal dispersion, and denser mitochondrial distribution compared with the original strain. Additionally, genome resequencing identified 57,216 single nucleotide polymorphisms (SNPs) in D24 relative to the original strain, including 4,068 non-synonymous SNPs. GO (Gene Ontology) and KEGG (Kyoto Encyclopedia of Genes and Genomes) enrichment indicated that the mutated genes were potentially involved in amino acid synthesis and metabolism, lipid metabolism, and others. These candidate mutations may be associated with the increased mycelial biomass and protein content. Collectively, this study has developed a novel high-yielding mutant of *G. frondosa*, which may serve as a promising material for future pharmaceutical and biotechnological applications.

**Supplementary information:**

The online version contains supplementary material available at https://doi.org/10.1186/s12896-026-01197-z.

## Introduction

Recently, continuous global population growth has led to a sharp rise in protein demand. However, despite being a complete protein source, animal-based production is unsustainable due to its high land use, significant greenhouse gas emissions, and associated health risks [1]. Therefore, there is an urgent need to develop efficient and environmentally friendly alternative protein sources. Microbial protein has thus emerged as a highly promising solution, offering advantages such as high productivity, low cost, and environmental friendliness, while providing the high-quality protein necessary for human physiological functions [2]. As an important member of eukaryotic microorganisms widely distributed worldwide, mushrooms have been used as medicine and food for a long time [3]. Among the 2000 characterized mushroom species, approximately 700 are identified as safe for human consumption, possessing nutritional and/or medicinal value [4]. *Grifola frondosa* (Dicks.: Fr.) SF Gray (Maitake), an important mushroom belonging to the family Grifolaceae Jülich, has received widespread attention due to its great edible, medicinal, and cosmetic potential [5, 6]. It is evident that the health benefits of *G. frondosa* are closely related to its abundant active components. A variety of valuable metabolites have been identified in this fungus [7, 8]. Notably, the protein content present in the mycelium or fruiting bodies of *G. frondosa* underlies its nutritional value and supports its consideration as a potential source of high-quality microbial protein [9]. Additionally, accumulated evidence has revealed the various pharmacological properties of *G. frondosa*, including anti-tumor [10], anti-obesity [11], anti-inflammatory [5], and others [12]. These findings collectively position *G. frondosa* as a significant reservoir of diverse bioactive constituents, leading to a marked increase in demand for this macrofungal species.

To meet the growing demand for *G. frondosa*, it is particularly important to establish a suitable cultivation process. With the gradual recognition of their extremely high economic value, the cultivation technology of mushrooms has been rapidly improved [13]. Nowadays, solid-state cultivation and submerged fermentation are the two main approaches used for mushroom production [14]. However, solid-state cultivation for mushroom production at the industrial level faces great challenges as its production efficiency is easily affected by various factors such as temperature, moisture, pH, heat transfer, etc [15]. Compared with solid-state cultivation, submerged fermentation—a technique for high-density cultivation of mushroom mycelium grown in liquid medium supplemented by aeration and agitation [16]—exhibits various advantages, including controllable process parameters, high production efficiency, short production time, and stable product quality. Thus, submerged fermentation technology has become a promising strategy for producing mushroom mycelium and its metabolites. In our previous study, liquid fermentation technology was also attempted to be used for the production of *G. frondosa*. However, preliminary results showed that slow hyphal growth and low protein yield have greatly limited its further application. Therefore, it is extremely important to select and breed novel *G. frondosa* strains with fast growth rates and high yields of proteins.

Nowadays, various traditional and modern breeding strategies (mutagenic breeding, protoplast fusion, genetically engineered breeding, mono-mono mating, genome shuffling, etc.) have been proposed to improve the efficiency of mushroom production [17–19]. Compared with other methods, artificial mutagenesis is considered a simpler, more effective, and low-cost strategy for strain improvement [20]. Mutagenesis breeding technology using physical or chemical mutagenic factors has been successfully used in the breeding of many fungi, including *Flammulina velutipes* [21], *Antrodia camphorata* [22], and *Volvariella volvacea* [23], etc. to improve their growth, active substance production, and adaptability to adverse external environments [24]. Another advantage of this strategy is that it does not require a detailed understanding of the target strain’s gene regulatory network. For filamentous fungal breeding, protoplasts are commonly used as the starting material. Due to the loss of the cell wall, protoplasts are more sensitive to external mutagens, facilitating the isolation of desirable mutants [25]. Hence, combining protoplasts with mutagenesis is widely regarded as an important means to increase the frequency and efficiency of mutations.

However, the preparation of protoplasts is a complex process, and its efficiency is easily affected by different factors [26]. Therefore, to improve the mutation efficiency of *G. frondosa*, the preparation process of protoplasts from *G. frondosa* was first optimized in the present study using a series of intelligent strategies. Subsequently, an attempt was made to breed a novel *G. frondosa* strain with a fast growth rate and high yield of proteins by protoplast-mediated diethyl sulfate (DES) mutagenesis. Furthermore, phenotype analysis, fermentation kinetics profiling, and genome resequencing of the wild (WT) and selected mutant were conducted. The findings presented herein provide candidate strain for the industrial application of *G. frondosa*.

## Materials and methods

### Fungal strain and cultivation

*G. frondosa* CGMCC 5.404, the parental strain used in this study, was bought from the China General Microbiological Culture Collection Center (Beijing, China). This fungal strain was cultured under conditions identical to those described in our prior study, utilizing the same medium and cultivation parameters [27].

### Preparation of protoplasts from *G*. *frondosa*

The protoplasts of *G. frondosa* were isolated according to a previous study with minor modifications [28]. Briefly, after seven days of growth, 0.1 g of *G. frondosa* mycelium was harvested by filtering the fermentation broth through four layers of gauze, then washed with sterile water and osmotic stabilizer, and subsequently incubated in 2% lywallzyme solution (dissolved in osmotic stabilizer, 1 mL) at 30 °C for 5 hours. Afterwards, the suspension was centrifuged at 3,000 r/min for 10 minutes, and the resulting precipitate was resuspended in osmotic stabilizer. Finally, the number of protoplasts was counted using a hemocytometer.

### Optimization of protoplasts preparation parameters

#### Single-factor test

To systematically evaluate the impact of different parameters (fungal age, lywallzyme concentration, enzymatic hydrolysis time, enzymatic temperature, and osmotic stabilizer) on protoplast yield, single-factor tests were employed. For fungal age, the mycelium grown for 5, 6, 7, 8, and 9 days were harvested and then subjected to hydrolysis using 2% lywallzyme dissolved in 0.6 mol/L mannitol at 30 °C. After five hours, the protoplast yield was quantified using a hemocytometer. For lywallzyme concentration, eight-day-old mycelia were hydrolyzed with 1%, 1.5%, 2%, 2.5%, and 3% lywallzyme solutions, respectively, while all other isolating parameters were kept constant as described above. Subsequently, the optimal enzymatic hydrolysis time was evaluated over a range of 2 to 6 hours, with the other parameters set as follows: fungal age of 8 days, lywallzyme concentration of 2%, enzymatic temperature of 30 °C, and 0.6 mol/L mannitol as the osmotic stabilizer. For enzymatic temperature, mycelia (8 days) were treated with 2% lywallzyme solution under various temperatures (26 °C, 28 °C, 30 °C, 32 °C,·34 °C) for 5 h, with all other isolation conditions maintained as described above. Finally, different osmotic stabilizers (MgSO_4_, KCl, sucrose, glucose, and mannitol) at a concentration of 0.6 mol/L were tested for protoplast isolation under fixed conditions (fungal age of 8 d, lywallzyme concentration of 2%, enzymatic hydrolysis time of 5 hours, and enzymatic temperature of 30 ℃). After quantifying protoplast yield, the optimal osmotic stabilizer was determined. All experiments were performed in triplicate.

#### Plackett-Burman design experiment

To investigate the influence of different parameters on protoplast production isolated from *G. frondosa*, a Plackett-Burman design (PBD) containing eight factors, each at two levels (−1 and +1), was implemented. According to this design matrix (Table 1), the *G. frondosa* protoplasts were isolated under different conditions, and the corresponding mean protoplast yield was calculated. Each experiment was conducted in triplicate. Then, analysis of variance (ANOVA) was performed to analyze the results according to the methods highlighted by Wang et al. [29].

**Table 1:** Plackett–Burman experimental design matrix and corresponding response values

Run	*X*_1_: Fungal age (d)	*X*_2_: Lywallzyme concentration (%)	*X*_3_: Enzymatic hydrolysis time (h)	*X*_4_: Enzymatic temperature (℃)	*X*_5_: Mannitol concentration (mol/L)	*X*_6_	*X*_7_	*X*_8_	*Y*: Protoplast yield (10^6^ protoplasts/g FW)
1	1 (10)	−1 (1)	1 (6)	−1 (28)	−1 (0.5)	−1	1	1	4.34 ± 0.04
2	1 (10)	1 (3)	−1 (4)	1 (32)	−1 (0.5)	−1	1	1	4.78 ± 0.13
3	−1 (6)	1 (3)	1 (6)	−1 (28)	1 (0.7)	−1	1	1	3.77 ± 0.06
4	1 (10)	−1 (1)	1 (6)	1 (32)	−1 (0.5)	1	1	1	5.23 ± 0.07
5	1 (10)	1 (3)	−1 (4)	1 (32)	1 (0.7)	−1	1	1	4.86 ± 0.04
6	1 (10)	1 (3)	1 (6)	−1 (28)	1 (0.7)	1	1	1	5.01 ± 0.09
7	−1 (6)	1 (3)	1 (6)	1 (32)	−1 (0.5)	1	1	1	4.57 ± 0.14
8	−1 (6)	−1 (1)	1 (6)	1 (32)	1 (0.7)	−1	1	1	3.98 ± 0.10
9	−1 (6)	−1 (1)	−1 (4)	1 (32)	1 (0.7)	1	1	1	2.98 ± 0.11
10	1 (10)	−1 (1)	−1 (4)	−1 (28)	1 (0.7)	1	1	1	3.56 ± 0.07
11	−1 (6)	1 (3)	−1 (4)	−1 (28)	−1 (0.5)	1	1	1	2.13 ± 0.02
12	−1 (6)	−1 (1)	−1 (4)	−1 (28)	−1 (0.5)	−1	1	1	1.92 ± 0.04

#### Box-Behnken design experiment

Based on the results of the PBD test, the optimal values of four main factors, namely, fungal age (A), lywallzyme concentration (B), enzymatic hydrolysis time (C), and enzymatic temperature (D), were determined using a four-factor, three-level Box-Behnken design (BBD), as illustrated in Table 2. Similarly, protoplasts were prepared according to the experimental design under each condition, and the average protoplast yield was used as the response variable. All above experiments were performed in triplicate. To analyze the results of the BBD experiment, two methods, namely response surface methodology (RSM) and the artificial neural network coupled with genetic algorithm approach (ANN-GA), were employed.

**Table 2:** Design matrix and results of the BBD experiment

Run	*A*: Fungal age (d)	*B*: Lywallzyme concentration (%)	*C*: Enzymatic hydrolysis time (h)	*D*: Enzymatic temperature (℃)	*Y*: Protoplast yield (10^6^ protoplasts/g FW)
1	−1 (6)	0 (2)	−1 (4)	0 (30)	3.99 ± 0.27
2	0 (8)	0 (2)	0 (5)	0 (30)	6.11 ± 0.12
3	0 (8)	0 (2)	1 (6)	−1 (28)	5.41 ± 0.26
4	0 (8)	−1 (1)	1 (6)	0 (30)	4.33 ± 0.26
5	0 (8)	0 (2)	−1 (4)	1 (32)	4.03 ± 0.29
6	1 (10)	0 (2)	0 (5)	−1 (28)	2.49 ± 0.23
7	1 (10)	0 (2)	−1 (4)	0 (30)	3.32 ± 0.17
8	−1 (6)	0 (2)	0 (5)	1 (32)	3.05 ± 0.40
9	1 (10)	0 (2)	0 (5)	1 (32)	2.47 ± 0.45
10	0 (8)	−1 (1)	−1 (4)	0 (30)	4.58 ± 0.25
11	0 (8)	1 (3)	1 (6)	0 (30)	3.90 ± 0.15
12	0 (8)	0 (2)	0 (5)	0 (30)	6.18 ± 0.08
13	0 (8)	−1 (1)	0 (5)	1 (32)	4.47 ± 0.13
14	0 (8)	1 (3)	−1 (4)	0 (30)	5.48 ± 0.11
15	1 (10)	−1 (1)	0 (5)	0 (30)	2.43 ± 0.21
16	−1 (6)	1 (3)	0 (5)	0 (30)	3.92 ± 0.44
17	0 (8)	−1 (1)	0 (5)	−1 (28)	4.64 ± 0.11
18	0 (8)	1 (3)	0 (5)	1 (32)	4.49 ± 0.16
19	−1 (6)	0 (2)	1 (6)	0 (30)	3.80 ± 0.26
20	1 (10)	1 (3)	0 (5)	0 (30)	2.74 ± 0.32
21	−1 (6)	0 (2)	0 (5)	−1 (28)	3.98 ± 0.33
22	0 (8)	0 (2)	−1 (4)	−1 (28)	5.46 ± 0.50
23	0 (8)	0 (2)	1 (6)	1 (32)	4.40 ± 0.43
24	1 (10)	0 (2)	1 (6)	0 (30)	2.75 ± 0.33
25	0 (8)	0 (2)	0 (5)	0 (30)	6.09 ± 0.08
26	0 (8)	1 (3)	0 (5)	−1 (28)	5.24 ± 0.41
27	0 (8)	0 (2)	0 (5)	0 (30)	6.25 ± 0.18
28	−1 (6)	−1 (1)	0 (5)	0 (30)	3.31 ± 0.31
29	0 (8)	0 (2)	0 (5)	0 (30)	6.50 ± 0.22

For RSM, to describe the relationship between these four factors and protoplast yield from *G. frondosa*, a multivariate quadratic regression (MQR) equation was established using the software Design-Expert 13. Furthermore, statistical analysis of this model was carried out referring to another article [30]. Additionally, feed-forward ANN model, an artificial intelligence learning tool inspired by the neuronal connections of the human brain, was also built using MATLAB software (R2006a, Math-Works Inc., Natick, MA, USA) to fit the data obtained from the BBD test [31]. In the present work, the architecture of the ANN consisted of an input layer containing four independent variables, a hidden layer containing multiple neurons, and an output layer (protoplast yield). However, due to the key role of neurons in the hidden layer in the performance of ANN, its optimal value was determined using the degree of approximation (*Da*) function, as described in previous studies [32]. In this formula, the constant was set to 5. After building a suitable ANN model, optimization was carried out using MATLAB software with a genetic algorithm to determine the optimal values of these four parameters. The parameters of the GA optimization process were set to be the same as those in a previous study [33]. Briefly, during the optimization process, the genetic algorithm was run according to the following parameter settings: the population adopted a double-vector form with a size of 20 and was randomly initialized. The stochastic uniform function was chosen for selection, with an elite count of 2 and a crossover fraction of 0.8, using a scattered crossover function. The migration fraction was 0.2, occurring every 20 generations.

#### Verification test and observation of protoplast morphology

The optimum preparation parameters predicted by RSM and ANN-GA were verified via verification tests. Briefly, the protoplasts were isolated from *G. frondosa* five times in parallel under the conditions predicted by RSM and ANN-GA, respectively, and the average protoplast yield was assessed. The performance of the RSM and ANN models was evaluated using several parameters: the coefficient of determination (*R*^2^), standard deviation (S.D.), mean square error (MSE), root mean square error (RMSE), chi square (**χ**^2^), and standard error of prediction (SEP) [34]. Furthermore, *G. frondosa* protoplasts were prepared under optimal conditions, and their morphology was photographed at 1, 3, and 5 hours using a microscope (TS2, Nikon, Japan), respectively. To assess the vitality of the isolated protoplasts, the protoplasts were subjected to trypan blue staining using the method described by Yu et al. [35].

### Regeneration of protoplasts

After protoplast isolation, the protoplast concentration was adjusted to 1 × 10^8^/mL using 0.6 mol/L mannitol. Three independent replicates were performed. Then, 0.1 mL of protoplast suspension was spread onto the fermentation medium containing 0.6 mol/L mannitol [36] and allowed to regenerate the cell wall at 25 ± 1 °C. After 10 days, the number of colonies grown on the medium was counted. Meanwhile, the protoplasts suspended in sterile water were also spread onto a fermentation medium without osmotic stabilizers (CONT group). Finally, the regeneration rate of protoplasts was calculated using the formula mentioned in Patil et al.’s study [37].

### Mutagenesis of *G*. *frondosa* using diethyl sulfate (DES)

#### DES Treatment for protoplasts isolated from *G. frondosa*

Protoplasts isolated from *G. frondosa* were gently resuspended in 0.6 mol/L mannitol (pH 7.0) and subjected to mutagenesis with DES at a final concentration of 2 mg/mL. Subsequently, the mutagenesis reaction was conducted under controlled conditions (25 °C, 135 r/min). Following DES exposure for varying durations (0, 2, 4, 6, 8, 10 min), protoplasts were immediately harvested, and the reaction was quenched by 200 μL of 25% (w/v) sodium thiosulfate solution. Furthermore, the protoplasts were spread onto the regeneration medium according to the method described in Sect. “Regeneration of protoplasts”, and the mortality rate for each duration was calculated. Three biological replicates were conducted for each treatment duration.

#### Screening of suitable mutants

To screen fast-growing mutants, the colonies of mutants and the wild strain (WT) were aseptically punched out using a 7-mm diameter sterile puncher and inoculated into equally sized wells on solid fermentation medium. The plates were then incubated at 25 °C for 15 days. Then, the colony diameters of each mutant and the parental strain were measured in triplicate. Following initial screening, mutant strains exhibiting significantly larger colony diameters than the parental strain were selected for secondary evaluation through submerged fermentation in Erlenmeyer flasks. Briefly, the selected strains were activated as described above. Then, the strains were inoculated into 100 mL Erlenmeyer flasks containing 40 mL fermentation medium [27]. The remaining cultivation parameters were set as follows: inoculation volume of 10% (v/v), temperature of 25 °C, agitation rate of 150 r/min, and a growth period of 8 days. After fermentation, the mycelium of each strain was harvested by filtration through four layers of sterile gauze and subsequently washed twice with deionized water. The collected mycelium was lyophilized using a lyophilizer (Genesis 2000 SQ, VIRTIS, USA) and ground into a fine powder (60 mesh). The dry mycelial weight was then measured, and the total protein content was determined. All the tests were carried out in triplicate.

#### Analyzing the genetic stability of mutant strains

Following the methodology of Lian et al., the selected mutants were subjected to ten consecutive subcultures at a 10% (v/v) inoculum to evaluate their genetic stability [38]. Additionally, the dry mycelial biomass and total protein content of the mutant strains were quantified at every second subculture passage. All the tests were carried out in triplicate.

### Protein extraction and content quantification

Proteins were extracted from the mycelium of selected strains and the wild strain using the ultrasound-assisted alkaline extraction method described by Zhang et al. with minor modifications [39]. Briefly, lyophilized mycelial powder (0.1 g) was homogenized in deionized water (9 mL), followed by pH adjustment to 10.0 using 0.1 mol/L NaOH solution. The resulting suspension was then subjected to ultrasonic treatment using an ultrasonic bath system (ultrasonic power: 400 W, ultrasonic temperature: 40 °C, ultrasonic time: 5 min). After ultrasonic treatment, the mixture was centrifuged (3000 r/min, 15 min, 4 ℃), and the supernatant was collected as the protein extract. Then, the protein concentration in the extract was determined using the Bradford kit according to the manufacturer’s instructions.

### Fermentation kinetics analysis of *G*. *frondosa* original strain and mutant strain D24

To compare the differences in mycelial growth kinetics and protein accumulation between the parental strain and mutant strain D24, growth curves and protein accumulation curves were constructed after batch fermentation over a period of 15 days. Briefly, the mutant strain D24 and the parental strain were cultivated in 250 mL Erlenmeyer flasks containing 100 mL fermentation medium. Other experimental conditions were set based on the above with minor modifications. The liquid fermentation medium consisted of glucose (25 g/L), peptone (12 g/L), yeast powder (10 g/L), potassium dihydrogen phosphate (2.5 g/L), magnesium sulfate (1.5 g/L), and vitamin B1 (0.05 g/L). The agitation rate was set at 150 r/min. During cultivation, the fermentation broth was harvested at 24-hour intervals to detect the pH, residual sugar concentration, mycelial biomass, and intracellular protein content in accordance with the methods described by other studies [40]. All above experiments were performed in triplicate. Origin 2022 software (Origin Lab, America) was used for nonlinear fitting of mycelial growth, protein accumulation, and substrate consumption.

Moreover, given the important role of fermentation kinetics in controlling microbial growth and enhancing product accumulation [41], the Logistic kinetic model was employed to describe the hyphal growth and protein accumulation of both *G. frondosa* strains. The calculation formulas of these three models are based on other research [42].

### Macroscopic and microscopic morphological observation of hyphae

The mycelia of *G. frondosa* mutant D24 and the WT strain were cultured according to the liquid fermentation method described above for 10 days. The fermentation broth was then filtered through four layers of sterile gauze to collect the mycelia. The harvested mycelia, along with the resuspension solution (mycelia were resuspended in 30 mL of phosphate-buffered saline (PBS)) were transferred into a 15 mm petri dish for morphological observation and image capture.

The hyphae of the *G. frondosa* mutant D24 and the parental strain were collected at two time points (4 and 6 days) during liquid fermentation. Then, scanning electron microscopy (SEM; Hitachi-SU8600, Hitachi High-Tech) and transmission electron microscopy (TEM; Hitachi-HT7800, Hitachi High-Tech) were used to observe the microscopic morphology of both strains [43]. These morphological observations were performed in triplicate.

### Whole-genome resequencing analysis of the *G*. *frondosa* D24 and the original strain

Mycelia of *G. frondosa* mutant D24 cultured for 6 days, along with those of the parental strain (cultured under the same conditions), were collected by filtration and immediately frozen in liquid nitrogen. Three independent biological replicates were performed for each strain. High-quality genomic DNA was extracted from the samples using a fungal genomic DNA extraction kit and sent to Annoroad Gene Technology (Beijing, China) for whole-genome resequencing. A DNA library with an insert size of 500 bp was constructed for each sample, and genome resequencing was performed using the Illumina HiSeq 2500 sequencing platform. Low-quality reads were filtered from the raw data to obtain high-quality reads (clean reads), which were then aligned to the *G. frondosa* reference genome (ASM168373v1, available at: https://www.ncbi.nlm.nih.gov/datasets/genome/GCA_001683735.1/) using BWA software (version 0.7.12). Single nucleotide polymorphisms (SNPs) and insertions/deletions (InDels) were detected using GATK software (v3.5) and then annotated using ANNOVAR. Subsequently, SNP sites with non-synonymous mutations in the mutant D24 relative to the parental strain were selected, and the corresponding genes were subjected to enrichment analysis against the KEGG (Kyoto Encyclopedia of Genes and Genomes) database and GO (Gene Ontology) databases.

### Statistical analysis

Results are expressed as the mean ± standard deviation (S.D.). Differences were considered statistically significant at *p* < 0.05 based on one-way ANOVA followed by Tukey’s post hoc test performed in GraphPad Prism 9.5.1 software (GraphPad Software, San Diego, California).

## Results and discussion

### Impact of different parameters on the generation of protoplasts in *G*. *frondosa*

As is well known, the establishment of standardized preparation parameters is essential for high-quality protoplast production. Due to interspecific variability in fungal cell wall composition, optimal protoplast isolation conditions must be determined for each organism. In this study, to evaluate the influence of different parameters on protoplast yield from *G. frondosa*, single-factor tests were introduced.

#### Fungal age of *G. frondosa*

**Fig. 1:**
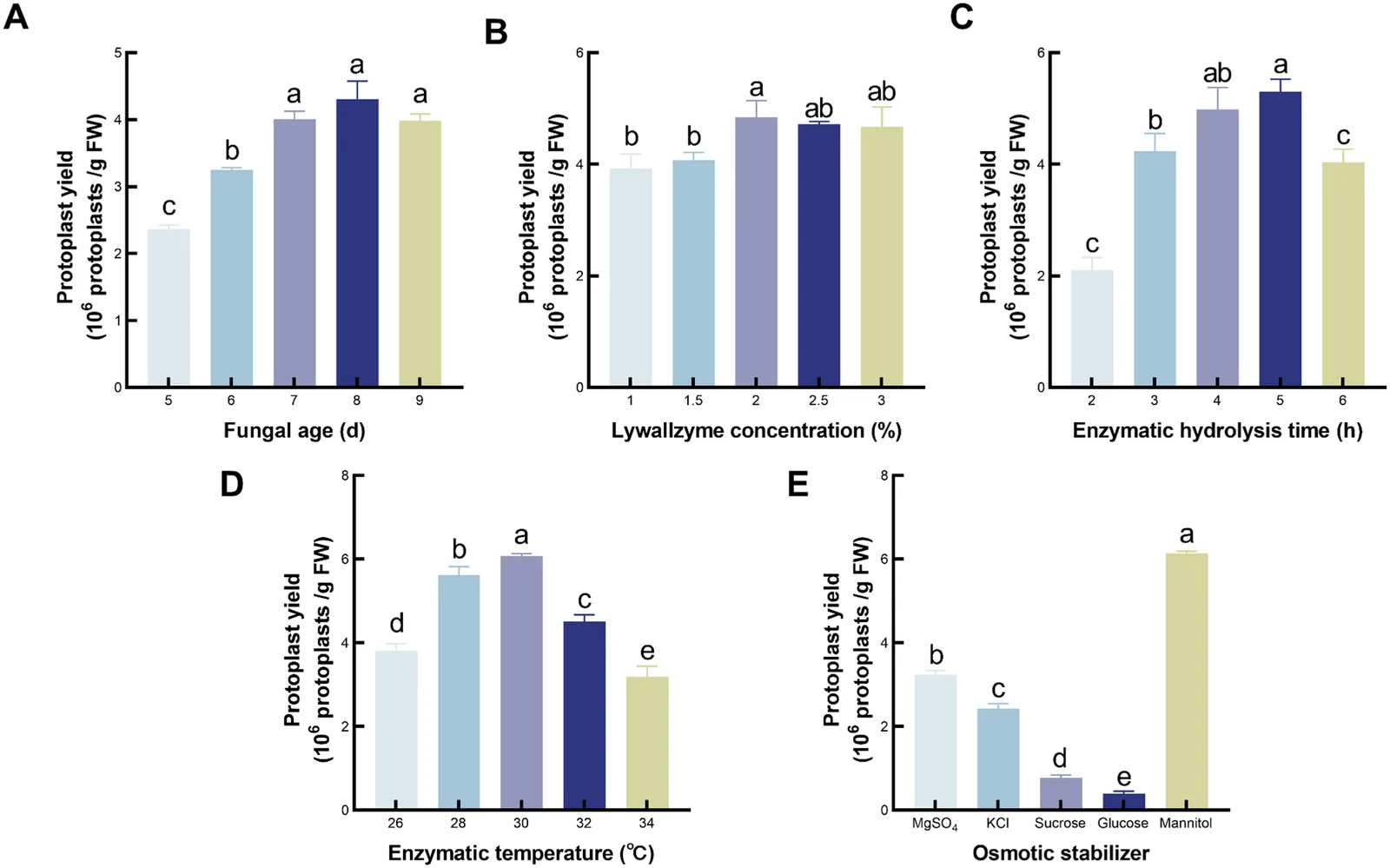
The influence of different factors on protoplast yield of *G. frondosa*. (**A**) Fungal age. (**B**) Lywallzyme concentration. (**C**) Enzymatic hydrolysis time. (**D**) Enzymatic temperature. (**E**) Type of osmotic stabilizer. Bar represents the mean ± S.D. of three replicate samples. Different lowercase letters in the figure indicate significant differences between groups (*p* < 0.05)

Hyphae of *G. frondosa* cultured for different durations were collected for protoplast isolation, and the corresponding results are presented in Fig. 1A. It is evident that the protoplast yield exhibited significant variation depending on the age of *G. frondosa*, indicating that fungi in different physiological states or growth stages have different levels of sensitivity to lytic enzymes. Although others found that young or exponentially growing hyphae are suitable for protoplasts isolation, the ideal fungal age varies among species [44]. For instance, the optimal ages for protoplast isolation from *Cordyceps militaris* [45], *Antrodia cinnamomea* [46], and *Lyophyllum decastes* [47] were 4 days, 30 hours, and 10 days, respectively. Moreover, previous results have indicated that both very young and aged hyphae are not conducive to the release and survival of protoplasts, which aligns with the findings presented here [45]. In the present work, results showed that as the age of *G. frondosa* increased from 5 to 8 days, the protoplast yield gradually increased. Referring to other articles, it is speculated that this may be due to the fact that the growth time was too short, resulting in less mycelium for enzymatic hydrolysis and therefore insufficient protoplasts [48]. In other words, although young *G. frondosa* mycelia possess thin cell walls that facilitate enzymatic hydrolysis, their inherently slow growth rate limits biomass accumulation, thereby reducing protoplast yield [47]. However, a reduction in protoplast amount was observed from hyphae cultured for more than 8 days, potentially attributable to cellular senescence, decreased mycelial vitality, increased cell wall thickness, and reduced susceptibility to lytic enzymes. This observation is consistent with the previous findings [49]. Hence, the proper fungal age of *G. frondosa* was selected as 8 days in the following experiments.

#### Concentration of lywallzyme

Numerous studies have revealed that the enzyme concentration is one of the limiting factors affecting protoplast formation. Thus, based on the above results, the proper lywallzyme concentration was determined. As illustrated in Fig. 1B, the highest protoplast yield was found in the 2% lywallzyme treatment group, and statistically significant differences were observed between this group and the hyphae treated with 1% or 1.5% lywallzyme (*p* < 0.05). Furthermore, increasing the lywallzyme concentration from 2% to 3% resulted in a slight reduction in *G. frondosa* protoplast yield (*p* > 0.05). Referring to previous studies, the decrease in protoplasts yield might be attributed to damage of cell membrane glycoproteins and nucleic acids [47]. Similar trends have also been observed in the preparation of protoplasts from other fungi, including *Gaeumannomyces tritici* [50] and *Hirsutella sinensis* [51]. The above data indicate that the suitable concentration of lywallzyme was 2%.

#### Enzymatic hydrolysis time

The impact of different enzymatic hydrolysis times (2, 3, 4, 5, 6 h) on protoplast yield was investigated. Fig. 1C showed that prolonged lywallzyme treatment resulted in a biphasic trend in protoplast yield, with an initial increase followed by a subsequent decline. The maximal protoplasts yield was achieved after 5 hours of incubation, demonstrating statistically significant differences compared to the 2-, 3-, and 6-hour treatment groups (*p* < 0.05). The results showed that with the increase of enzymatic hydrolysis time, the cell wall of the mycelium gradually lysed, thereby enhancing protoplast yield. However, the decrease in the amount of protoplasts caused by further prolonged enzymatic hydrolysis might be related to its destructive effect on the cell membrane structure of protoplasts [51]. These findings were in accordance with other previous studies. For instance, after enzymatic hydrolysis of *Ganoderma boninense* for different hours (1, 1.5, 2, 2.5 hours), the 2-hour treatment group showed significantly higher protoplast amounts [52]. Additionally, Ishikawa and coworkers found that the effect of enzymatic hydrolysis time on fungal protoplasts production was also related to the enzyme concentration [53]. They found that the trend of protoplast production varied with the extension of enzymatic hydrolysis time when the concentration of lysing enzyme was 10, 20, and 30 mg/mL. Hence, the enzymatic hydrolysis time used in the following single-factor tests was selected as 5 h.

#### Enzymatic temperature

It is widely known that temperature is a key factor affecting the rate of enzymatic reactions. Therefore, it is undoubted that finding the optimal enzymatic temperature is particularly important for enhancing the protoplast yield. Results showed that the highest protoplasts yield (6.08 × 10^6^ protoplasts/g FW) was obtained at 30 °C, which was markedly higher than at other incubation temperatures tested (Fig. 1D, *p* < 0.05). This change might be explained as an increase in enzyme activity and reaction rate with rising temperature, leading to an increase in the amount of protoplasts. However, once the temperature exceeded 30 °C, the rate of enzymatic hydrolysis by lywallzyme decreased. Thus, the suitable enzymatic temperature for protoplast preparation from *G. frondosa* was 30 °C.

#### Osmotic stabilizers

Suitable osmotic stabilizers are particularly important for preventing osmotic lysis of protoplasts and maintaining their stability [54]. To date, many osmotic stabilizers have been used in the preparation of plant and microbial protoplasts, such as inorganic salts, sugars, etc. [55]. Indeed, the suitable osmotic stabilizer for different microorganisms is not entirely consistent. For *Metarhizium anisopliae* var. Majus, 0.7 mol/L KCl was demonstrated to be the optimal osmotic stabilizer [56]. Taking *Sclerotium rolfsii* as another example, Fariña et al. found that the highest protoplast yield was observed in the presence of MgSO_4_ as an osmotic stabilizer [57]. In the present work, five osmotic stabilizers were employed to isolate the protoplasts from the target fungus. Significant differences were observed in the number of obtained protoplasts using different osmotic stabilizers (Fig. 1E). Clearly, 0.6 M mannitol, as a widely used stabilizer, produced the highest protoplast yield of up to 6.14 × 10^6^ protoplasts/g FW, followed by MgSO_4_, KCl, and sucrose, while glucose was the least effective stabilizer for *G. frondosa* protoplasts. Similarly, other researchers have found that mannitol is suitable for the isolation of protoplasts from many microbial species, including *Ganoderma lucidum* [58], *Cordyceps cicadae* [59], etc. Therefore, mannitol was employed to be used for subsequent experiments.

### Selection of effective variables obviously influences protoplast yield from *G*. *frondosa*

To evaluate the relative influence of various parameters on *G. frondosa* protoplast production, a PBD consisting of eight factors was introduced, which is a widely used tool for identifying key factors significantly affecting system performance [60]. As depicted in Table 1, the number of protoplasts obtained under different preparation conditions varied, ranging from 1.92 × 10^6^ to 5.23 × 10^6^ protoplasts/g FW. Minitab 22 software was then used to perform regression analysis and ANOVA, as summarized in Table S1. To model the relationship between these factors and protoplast yield, the following regression model was established.

1 $$\eqalign{ Y &= {\rm{ }}3.9328{\rm{ }} + {\rm{ }}0.7025{X_1} + {\rm{ }}0.2592{X_2} + {\rm{ }}0.5558{X_3} + {\rm{ }}0.4725{X_4} \cr & + {\rm{ }}0.0992{X_5} - {\rm{ }}0.0142{X_6} - {\rm{ }}0.0208{X_7} - {\rm{ }}0.0575{X_8} \cr} $$

Data showed that the *F* value and *p* value of the established model were 55.86 and 0.004, respectively, indicating that the model was significant. Moreover, a high *R*^2^ (0.993) and adjusted *R*^2^ (0.976) were obtained, suggesting that the data are well explained by the model. This indicates that only 0.7% of variability is not explained by the above model, further supporting the model’s reliability. Based on the *p*-values of individual factors, the order of their impact on the response value was: *X*_1_ (*p* = 0.001) > *X*_3_ (*p* = 0.002) = *X*_4_ (*p* = 0.002) > *X*_2_ (*p* = 0.014) > *X*_5_ (*p* = 0.140) > *X*_8_ (*p* = 0.332) > *X*_7_ (*p* = 0.704) > *X*_6_ (*p* = 0.794). Among the tested factors, four variables (*X*_1_, *X*_3_, *X*_4_, and *X*_2_) with lower *p* values (*p* < 0.05) were identified as main factors markedly affecting the amount of protoplasts. Additionally, the positive coefficients of these four factors indicate that higher fungal age, enzymatic hydrolysis time, enzymatic temperature, and lywallzyme concentration were beneficial for enhancing protoplast yield. The relative contribution of each factor on the target variable was also visually displayed in Fig. S1, which showed that fungal age (*X*_1_) had the most significant impact on the yield of protoplasts isolated, followed by enzymatic hydrolysis time (*X*_3_), enzymatic temperature (*X*_4_), and lywallzyme concentration (*X*_2_). Taken together, the optimal values of these four parameters were further optimized.

### Optimizing the suitable values of four main parameters using RSM and ANN-GA

As mentioned above, a BBD design with 29 trials was introduced to obtain the optimum values of fungal age, enzymatic hydrolysis time, enzymatic temperature, and lywallzyme concentration. The average amount of protoplasts isolated under each preparation condition in the design matrix is listed in Table 2. The maximum protoplast yield reached 6.50 × 10^6^ protoplasts/g FW, while the lowest yield was 2.43 × 10^6^ protoplasts/g FW, indicating the necessary of optimizing the preparation conditions for protoplast isolation from *G. frondosa*. Furthermore, two methodologies were employed to analyze these data, namely RSM and ANN-GA.

#### RSM analysis

To understand the interactive effects of selected parameters on protoplast yield, Design Expert 13 software was introduced, and a quadratic regression equation with four variables was established as follows:

2 $$\eqalign{ Y & = 6.226 - 0.488A{\rm{ }} + {\rm{ }}0.168B - {\rm{ }}0.189C - {\rm{ }}0.359D - {\rm{ }}0.075AB \cr &- {\rm{ }}0.095AC + {\rm{ }}0.228AD - {\rm{ }}0.333BC - {\rm{ }}0.145BD+ {\rm{ }}0.105CD \cr &- {\rm{ }}2.277A^{2}{\rm{ }} - {\rm{ }}0.867B^{2} - {\rm{ }}0.627C^{2}{\rm{ }} - {\rm{ }}0.792D^{2} \cr} $$

**Fig. 2:**
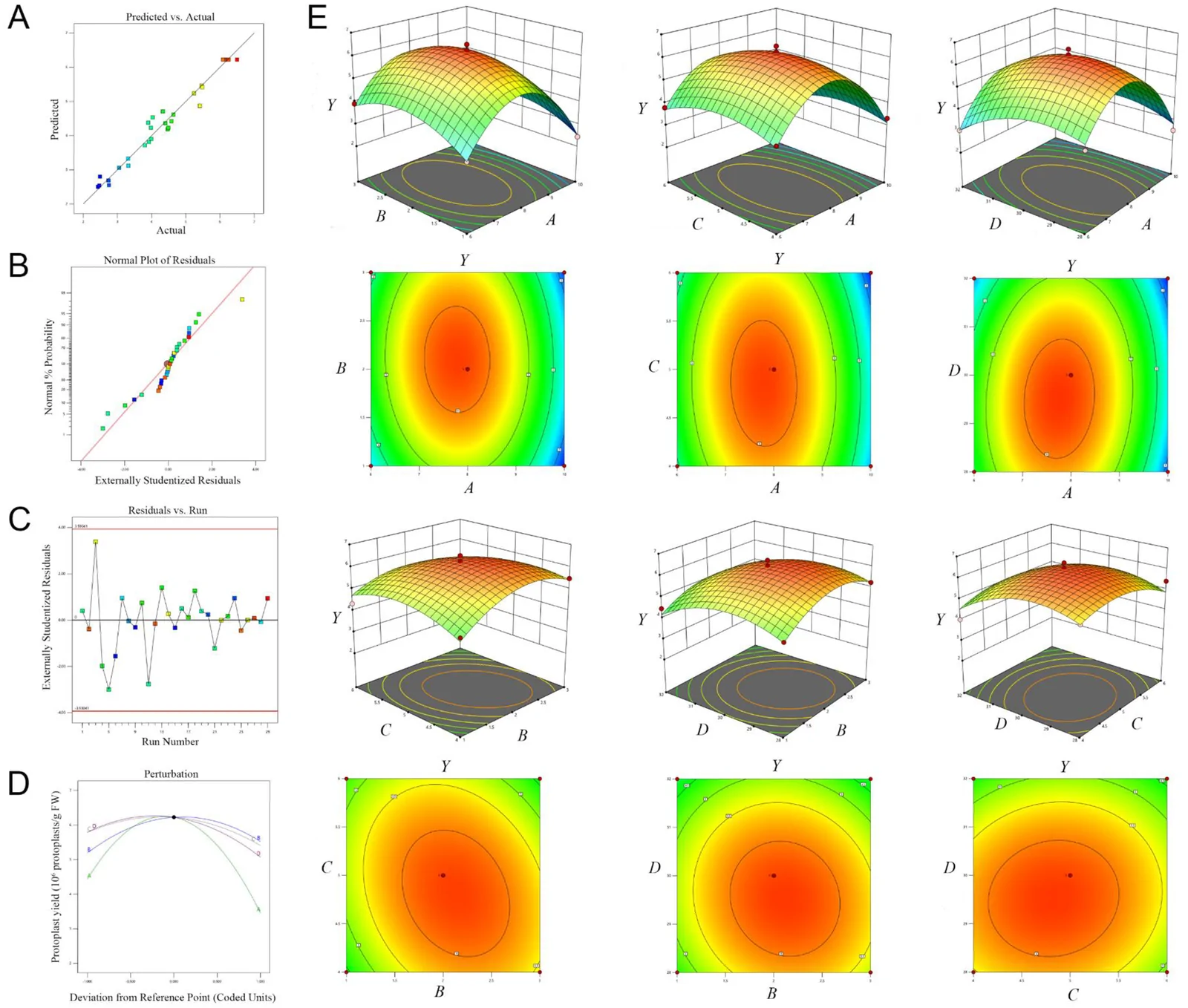
Results of the response surface analysis. (**A**) Comparison chart between the predicted and observed values. (**B**) Plot used to describe the relationship between the normal probability and internally studentized residuals. (**C**) Diagram of the relationship between studentized residuals and run number. (**D**) Perturbation plot. (**E**) Response surface and contour plots

ANOVA was also performed to evaluate the predictive performance of the developed model, a common method in regression analysis [61]. The ANOVA results (Table 3) showed that the established model was significant and suitable for fitting the experimental data, as indicated by a small *p* value (*p* < 0.0001) and a high *F*-value (*F* = 28.05). Meanwhile, very close adjusted *R*^2^ (0.9312) and *R*^2^ (0.9656) of the model were observed, and both were close to 1, depicting that little noise could not be understood with this model. On the other hand, the significance of the model’s lack of fit term was noted, which is a widely used indicator to evaluate the reliability of a model by comparing the residual error with pure errors [27]. In the present study, the lack of fit was considered insignificant (*p* value > 0.05), suggesting the adequacy of the established model. Correlation analysis between the values predicted by the MQR model and the actual protoplast yield was performed, and the results are plotted in Fig. 2A. A straight line was found in this chart, illustrating the adequacy of the developed model for data fitting. Furthermore, the reliability of the model predictions was confirmed by the results displayed in Figs. 2B and 2C. Specifically, a straight line was observed in the normal probability versus externally studentized residuals (Fig. 2B). With regard to the diagram of the relationship between studentized residuals and run number, no point exceeded the red line, suggesting that no potential error fell outside the allowed range (Fig. 2C). Overall, these data indicate that the developed MQR model is feasible for fitting and predicting the experimental results.

**Table 3:** Analysis of variance for the model obtained in response surface analysis

Source	Sum of squares	df	Mean square	*F*-value	*p*-value
Model	41.5400	14	2.9700	28.0500	<0.0001
*A*	2.8500	1	2.8500	26.9600	0.0001
*B*	0.3367	1	0.3367	3.1800	0.0961
*C*	0.4294	1	0.4294	4.0600	0.0635
*D*	1.5500	1	1.5500	14.6300	0.0019
*AB*	0.0225	1	0.0225	0.2127	0.6517
*AC*	0.0361	1	0.0361	0.3413	0.5684
*AD*	0.2070	1	0.2070	1.9600	0.1836
*BC*	0.4422	1	0.4422	4.1800	0.0602
*BD*	0.0841	1	0.0841	0.7950	0.3877
*CD*	0.0441	1	0.0441	0.4169	0.5289
*A*^2^	33.6200	1	33.6200	317.8500	<0.0001
*B*^2^	4.8700	1	4.8700	46.0700	<0.0001
*C*^2^	2.5500	1	2.5500	24.0900	0.0002
*D*^2^	4.0700	1	4.0700	38.4400	<0.0001
Residual	1.4800	14	0.1058		
Lack of fit	1.3700	10	0.1371	5.0000	0.0674
Pure error	0.1097	4	0.0274		
Cor total	43.0300	28

As depicted in Table 3, six terms (*A*, *D*, *A*^2^, *B*^2^, *C*^2^, *D*^2^) had *p* values less than 0.05, indicating their significant impacts on protoplast yield from *G. frondosa*. Meanwhile, to intuitively know the effects of the four investigated parameters on protoplast yield, a perturbation plot was generated using software Design-Expert 13. As shown in Fig. 2D, parameters *B*, *C*, and *D* exhibited similar effects on the target response variable, whereas variable *A* had a significantly greater impact. These findings suggest that protoplast amount was primarily influenced by fungal age. As variable A increased from low to high levels, protoplast yield showed a trend of first increasing and then decreasing, consistent with the phenomenon found in single-factor tests. Additionally, the *p* values of all interaction terms were greater than 0.05, indicating that the interaction between factors was not significant. Response surface plots and contour plots were also established to visually examine the interaction among the four selected variables (Fig. 2E), further confirming the absence of significant interactions.

Then, the optimal preparation conditions for *G. frondosa*’s protoplasts predicted by the MQR model were obtained as follows: fungal age 7.8 days, lywallzyme concentration 2.2%, enzymatic hydrolysis time 4.8 hours, enzymatic temperature 29.5 °C, and mannitol concentration 0.6 mol/L. The maximum protoplast yield predicted by the model was 6.338 × 10^6^ protoplasts/g FW, which had a small error (1.45 %) compared to the actual yield of protoplasts (6.43 × 10^6^ protoplasts/g FW) prepared under the above optimal conditions.

#### Development of the ANN model and optimization using GA

Numerous studies have mentioned that, compared to RSM, ANN coupled with GA have demonstrated higher prediction accuracy in many optimization processes [62]. Due to its superior parameter generalization and remarkable validation accuracy, ANN-GA has gradually gained popularity and is widely used as a powerful hybrid intelligent technique in various fields, such as food, pharmaceuticals, engineering, etc. [63, 64]. Therefore, ANN-GA was also employed to further optimize the suitable values of four variables.

**Fig. 3:**
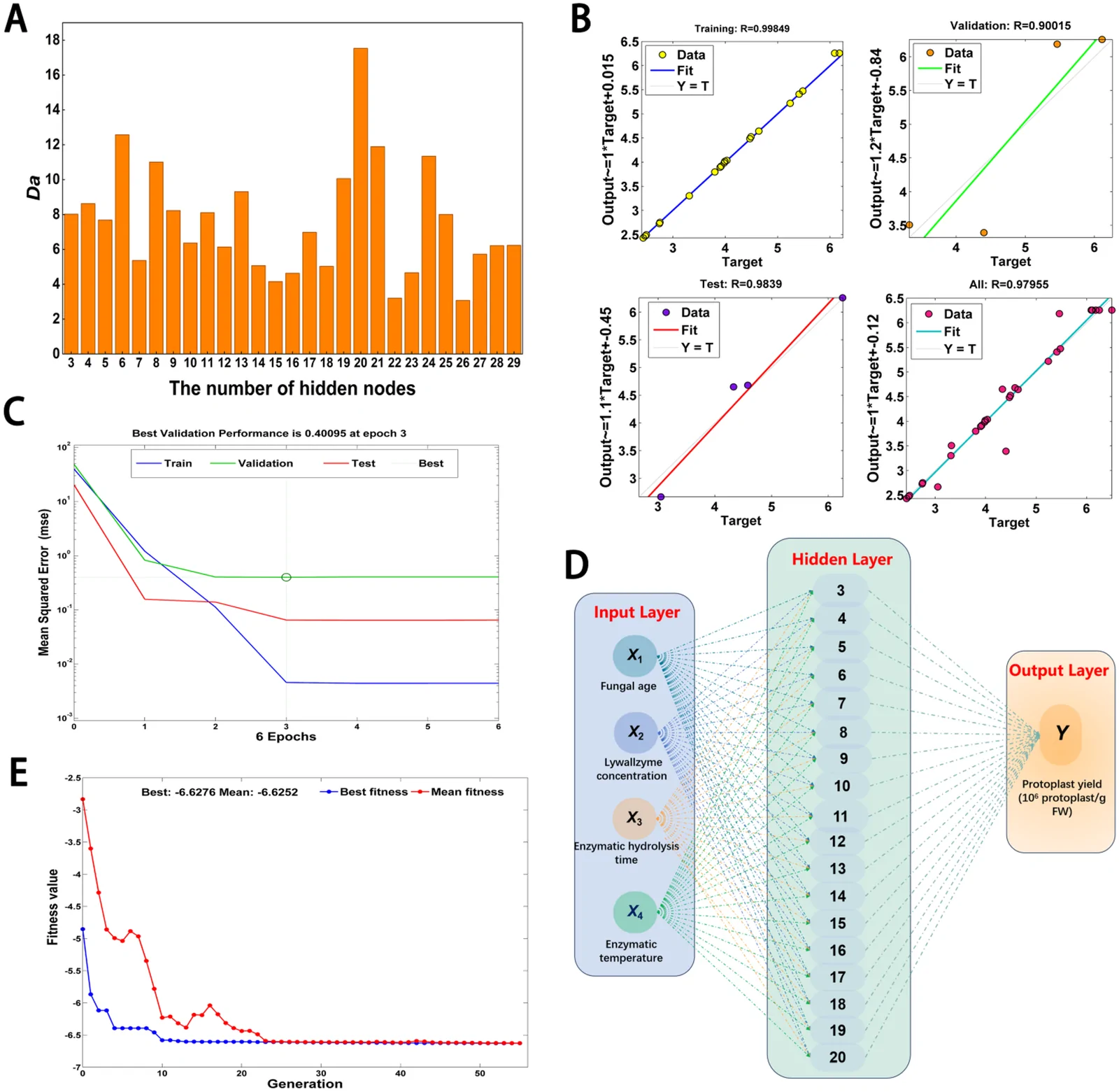
Optimization of the parameters for *G. frondosa* protoplasts preparation using ANN coupled with GA. (**A**) Screening of the suitable number of neurons in the hidden layer. (**B**) Regression analysis between actual and target data. (**C**) Training performance of the selected ANN model. (**D**) The architecture topology of the selected ANN model (4–20–1) used in the present study. (**E**) The change in fitness value with the increase in generation

In this study, a feed-forward ANN network with three layers (input layer, hidden layer, output layer) was developed, in which the output layer was the protoplasts yield from *G. frondosa*, and four investigated variables were used as four input layers. To select the appropriate number of hidden neurons, the ANN network was trained with varying numbers of neurons in the hidden layer (ranging from 3 to 29), and the corresponding *Da* value was calculated. As depicted in Fig. 3A, the suitable number of hidden neurons was determined to be 20, as it yielded the maximum *Da*. The ANN network with 20 neurons in the hidden layer exhibited a highly *R*^2^ value (Fig. 3B). Additionally, the analysis of mean square error against iteration numbers revealed that the model reached a lower MSE (0.40095) after three iterations (Fig. 3C). Hence, the best-performing ANN model (Fig. 3D) was established with a topological architecture of 4–20–1.

Subsequently, GA was further used to find the optimal values for the four investigated factors using the fitness function. It can be obviously observed that as the number of generations increased, the fitness value gradually decreased and eventually remained almost unchanged after the 23rd generation, and the mean fitness value was achieved to −6.6252 (Fig. 3E). After prediction, the maximum amount of protoplasts was expected to reach 6.6275 × 10^6^ protoplasts/g FW under optimized conditions (7.9 d of fungal age, 2.1% of lywallzyme concentration, 5.2 h of enzymatic hydrolysis time, 30 °C of enzymatic temperature, and 0.6 mol/L of mannitol concentration). To verify this prediction, protoplasts were isolated from *G. fronodosa* under the optimal conditions predicted. The results showed that the average production of protoplasts obtained from five validation experiments was 6.573 × 10^6^ protoplasts/g FW, which deviated from the predicted maximum response value by only 0.822%.

#### Comparison of RSM and ANN

To evaluate the fitting and predictive efficiency of the two optimization strategies, a total of six parameters were employed, including the coefficient of determination (*R*^2^), standard deviation (S.D., %), mean square error (MSE), root mean square error (RMSE), chi square (**χ**^2^), and standard error of prediction (SEP, %). As illustrated in Table S2, the *R*^2^ values of the two models were both higher than 0.95, indicating a high consistency between the actual results and the values predicted by these two models. Nevertheless, the *R*^2^ value of RSM was higher than that of ANN, suggesting superior accuracy of RSM. Additionally, lower MSE, S.D., RMSE, **χ**^2^, and SEP values were observed for the RSM model compared to the ANN model, indicating that the predictive ability of the RSM model was superior to that of the ANN model in this study [65]. The predictive performance of well-established RSM models is known to depend on factors like experimental scale and treatment type. Generally, RSM can sometimes provide relatively accurate predictions even with a small number of experiments, whereas ANN performs robustly regardless of the data size. Given the limited dataset (*n* = 29) used in this work, RSM proved to be more effective, although ANN still yielded efficient predictions. Similar findings have been reported by Zeng et al. (2020) and Naderloo (2020) when comparing the performance of RSM and ANN models [66, 67].

**Fig. 4:**
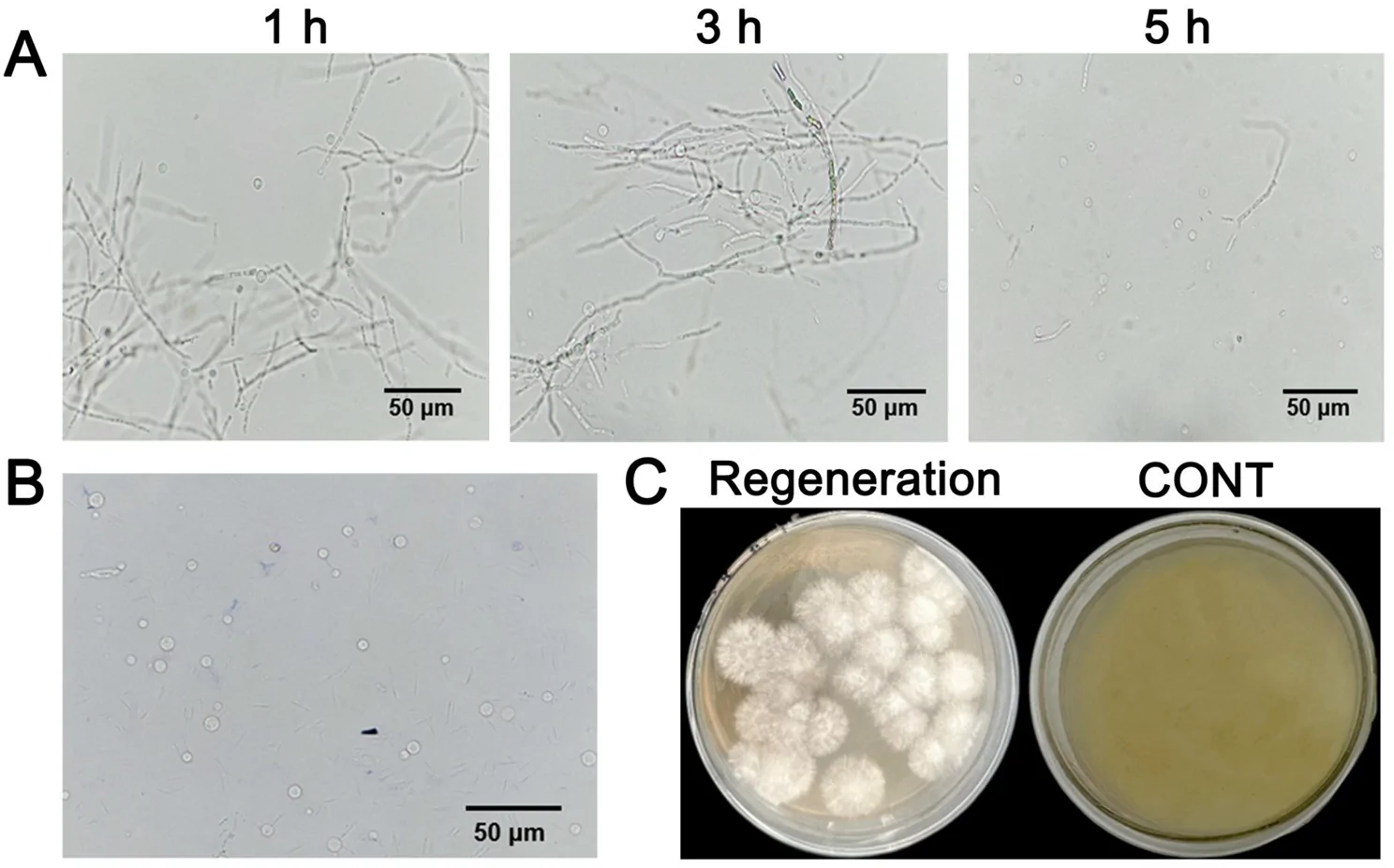
Phenotypic observation of protoplast generation and regeneration in *G. frondosa*. (**A**) Morphological changes of protoplasts at various time points during enzymatic digestion. (**B**) Evaluation of protoplast viability using trypan blue staining. (**C**) Regeneration phenotype of protoplasts derived from *G. frondosa*

Moreover, the protoplast morphology was observed and photographed during the enzymatic hydrolysis process. As is well established, specific enzymes can recognize and degrade cell wall components, such as chitin and cellulose, leading to cell wall disruption. In the absence of cell wall support and constraint, protoplasts naturally assume a spherical morphology. Obviously, as the enzymatic hydrolysis time increased, the hyphae were progressively hydrolyzed, leading to a corresponding increase in spherical protoplasts and a reduction in hyphal fragments (Fig. 4A). To assess protoplast viability, trypan blue staining was employed, as this dye selectively penetrated non-viable cells. As depicted in Fig. 4B, the protoplasts exhibited larger, rounded morphology with minimal trypan blue staining, indicating high viability and ensuring optimal regeneration potential. On the basis of the above results, the prepared protoplasts were allowed to regenerate cell walls in regeneration medium, and their regeneration rate was calculated to be 3.62 × 10^−1^% (Fig. 4C). Nevertheless, despite the fact that low protoplast regeneration rates have been documented in previous studies [68], the regeneration rate obtained in this study appears to be relatively low in comparison with previously reported values. As reported, the regeneration rates of protoplasts for *C*. *militaris* [45] and *G*. *lucidum* [58] reached 36.5% and 3%, respectively, both of which were higher than the rate observed in the present work. It is possible that the nutrient composition, osmotic stabilizer, and pH of regeneration medium may account for this discrepancy, warranting further optimization [46]. For instance, the protoplast regeneration rate of *L. decastes* differed significantly among the five regeneration media tested [47]. In the case of *H*. *sinensis*, for instance, the protoplast regeneration rate was below 1% when mannitol served as the osmotic stabilizer in the regeneration medium [51]. Meanwhile, previous study have found that the agar concentration in the solid medium was associated with the protoplast regeneration rate [51]. Additionally, referring to other research [69], the conditions employed during protoplast preparation, such as enzyme composition, digestion time, and osmotic stabilizer, may also influence the regeneration efficiency and could partially explain the low regeneration rate observed in *G. frondosa*. Therefore, further optimization of the regeneration conditions remains a key priority for future work.

### Effect of DES treatment on the survival rate of protoplasts prepared from *G*. *frondosa*

**Fig. 5:**
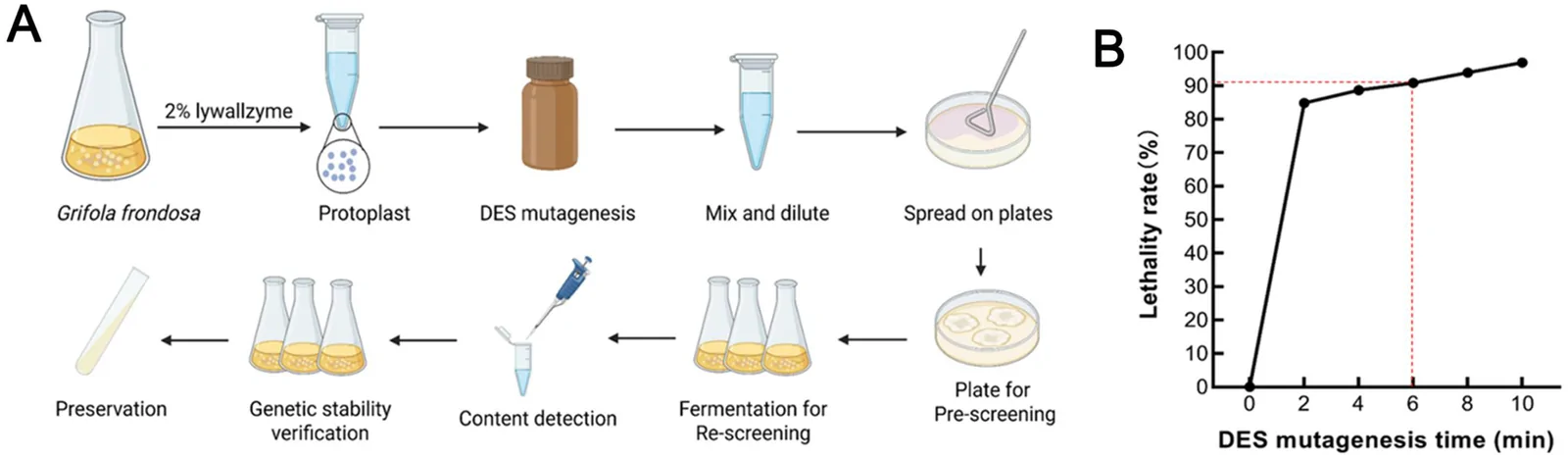
DES Mutagenesis of protoplasts from *G. frondosa*. (**A**) Flow chart of DES mutagenesis and screening. (**B**) Effect of DES exposure duration on the lethality rate of protoplasts prepared from *G. frondosa*

Although numerous *G. frondosa* strains have been isolated, strain CGMCC 5.404 was selected as the parental strain in this study, a choice also made in several previous investigations [70–72]. Moreover, its hypolipidemic and antioxidant activities have been demonstrated in our previous work and other studies [27, 72]. These findings suggest that this strain may have some potential for future application. Thus, with the aim of improving its biomass yield, the effect of DES exposure time on protoplast viability was examined, as a relatively low lethality rate is generally considered favorable for effective mutant screening [73]. The flow chart of DES mutagenesis is shown in Fig. 5A. As depicted in Fig. 5B, DES exposure induced rapid protoplast lethality, with mortality exceeding 80% within 2 minutes, and showing a time-dependent increase. At 6 minutes of exposure, the mortality rate reached over 90%. However, prolonging the mutagenesis time further increased the lethality rate of protoplasts, resulting in excessive protoplast death that hinders effective mutant screening. Referring to previous studies, a mutagenesis time of 6 minutes was selected for subsequent screening [74].

### Breeding *G*. *frondosa* with enhanced biomass and protein yield using DES mutagenesis

#### Preliminary screening of suitable *G. frondosa* mutant strains

**Fig. 6:**
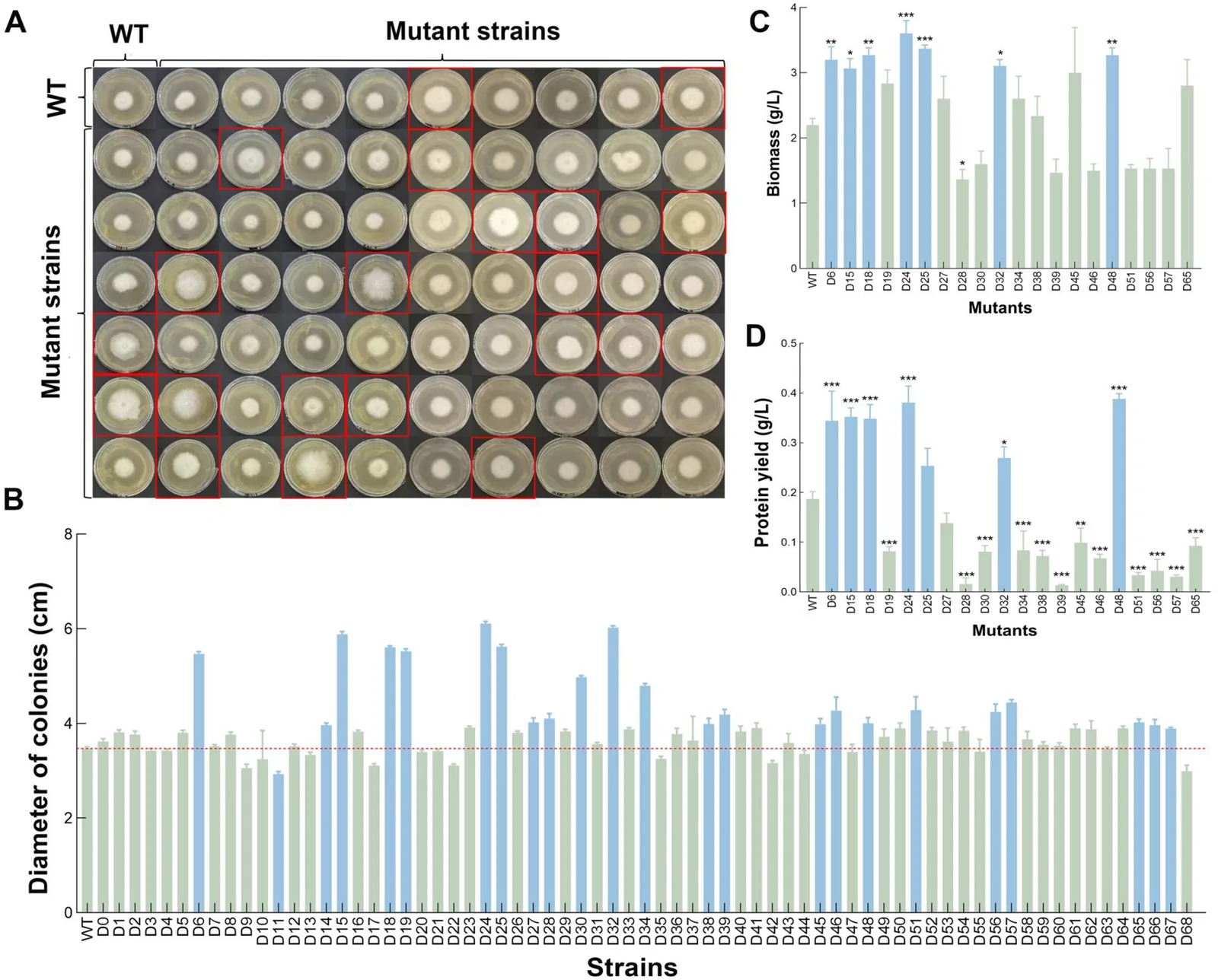
Preliminary screening of *G. frondosa* mutant strains. (**A**) Growth phenotypes of *G. frondosa* mutant strains. (**B**) Statistical chart of colony diameters of *G. frondosa* mutant strains. Bar represents the mean ± S.D. of three replicate measurements. All the colony diameters of mutants and the parental strain were measured in triplicate. The red dashed line indicates the mycelial dry weight of the WT strain. For the light blue bars, the colony diameter of the mutant strain shows a significant difference compared to the WT strain (*p* < 0.05). (**C** and **D**) the biomass yield (**C**) and the protein production (**D**) of *G. frondosa* mutant strains. Bar represents the mean ± S.D. of three replicate samples. Asterisks denote statistically significant differences compared with the WT strain (*p < 0.05, **p < 0.01, ***p < 0.001)

As mentioned above, the protoplasts treated with DES for 6 min were plated on the regeneration medium, and a total of 69 single colonies were selected from the plates. Then, these strains were further screened based on the colony diameter as an indirect indicator of growth rate (Fig. 6A). Notably, many mutant strains exhibited larger colony sizes compared to the wild-type strain, with the most prominent examples highlighted by red squares. Furthermore, quantitative measurements of colony diameter confirmed these morphological differences. As illustrated in Fig. 6B, there are 52 strains with colony diameters larger than the WT strain, of which 23 (indicated by light blue labeling, excluding D11) have statistically significant differences from the WT strain (*p* < 0.05). Among these mutants, 20 strains with the top 20 largest colony sizes were selected for subsequent screening.

#### The biomass and protein production of selected *G. frondosa* mutant strains

The mutant strains selected from the preliminary screening were further evaluated using liquid fermentation in flasks for 8 days, and their mycelial biomass and protein yield were determined (Fig. 6C and D). Regarding mycelial biomass, in comparison with the WT strain, obvious differences were found in 8 mutant strains, including 7 positive mutant strains and 1 negative mutant strain (Fig. 6C). Among these, the D24 mutant strain exhibited the highest mycelial biomass, which was 1.64 times that of the original strain. Moreover, Fig. 6D depicts the protein production of each mutant strain. There were 6 mutant strains with significantly higher protein production than that of the WT strain (*p* < 0.05). Specifically, the protein production of mutant strains D6, D15, D18, D24, D32, and D48 increased by 78.95%, 84.21%, 84.21%, 100%, 42.11%, and 105.26%, respectively, compared with the parental strain. The highest protein production (0.39 g/L) was observed in the mutant strain D48, which was significantly higher than that of the WT strain (0.19 g/L). Based on comprehensive comparative analysis of mycelial biomass and protein production among the different mutant strains, seven mutant strains (D6, D15, D18, D24, D25, D32, and D48) were selected for further investigation of their genetic stability.

#### Genetic stability analysis of candidate *G. frondosa* mutant strains and antagonistic reaction

To assess the genetic stability of candidate mutant strains, both the selected mutants and the WT strain were subjected to 10 sequential subcultures, with mycelial biomass and protein yield quantified at alternating generations. As summarized in Table 4, mutant strain D24 demonstrated superior mycelial biomass and protein accumulation while maintaining genetic stability, as evidenced by consistent production yields across multiple subculture generations (*p* > 0.05). Therefore, the mutant strain D24 was selected to use in further studies. Notably, the mycelial biomass of D24 obtained in this work is comparable to or higher than those reported in several liquid fermentation studies of *G. frondosa*. For instance, Sim et al. reported a mycelial biomass of 1.3 g/L for *G. frondosa* after liquid fermentation for 14 days [75]. Taking *G. frondosa* CCRC36911 as an example, a higher mycelial biomass of 2.32 g/L was achieved when yeast extract was used as the nitrogen source [76]. Conversely, some studies have reported higher mycelial biomass of *G. frondosa* through liquid fermentation compared to that of mutant D24. Cui et al. Obtained a mycelial biomass of *G. frondosa* GF9801 as high as 17.61 g/L after optimizing the liquid medium [77]. Beyond flask culture, fermenter-based production of *G. frondosa* mycelium has also been explored. Shih et al. found that the mycelial dry weight of *G. frondosa* was enhanced to 8.23 g/L in a 5-L fermenter using glucose fed-batch fermentation [78]. In addition, a mycelial dry weight as high as 18 g/L was achieved for *G. frondosa* mutant strain (M270) after fermentation in a 20-L bioreactor [79]. However, compared to mycelial biomass production of *G. frondosa* via liquid fermentation, protein production remains comparatively underexplored. In the study by Sim et al., for example, the soluble protein yield was 283.6 µg/mL under liquid fermentation conditions [75]. It is suspected that the observed differences in mycelial yield are closely associated with strain-specific properties and environmental factors. Therefore, further efforts are warranted to optimize the large-scale fermentation process for the mutant strain D24 used in the present study.

**Table 4:** Genetic stability analysis of *G. frondosa* mutant strains

Production		F0	F2	F4	F6	F8	F10
Biomass (g·L^−1^)	WT	2.20 ± 0.01	2.27 ± 0.01	2.30 ± 0.01	2.27 ± 0.01	2.23 ± 0.01	2.23 ± 0.01
	D6	3.53 ± 0.06***	3.53 ± 0.00***	3.67 ± 0.01***	3.53 ± 0.02***	3.67 ± 0.03***	3.60 ± 0.01***
	D15	3.07 ± 0.02**	3.03 ± 0.00***	3.15 ± 0.01***	2.55 ± 0.01	2.60 ± 0.01	2.53 ± 0.01*
	D18	3.27 ± 0.01**	3.03 ± 0.02***	3.10 ± 0.01***	3.23 ± 0.02***	3.15 ± 0.02***	3.10 ± 0.01***
	**D24**	3.60 ± 0.02***	3.5 ± 0.01***	3.60 ± 0.01***	3.5 ± 0.01***	3.55 ± 0.02***	3.63 ± 0.02***
	D25	3.37 ± 0.00***	3.1 ± 0.01***	3.03 ± 0.01***	2.23 ± 0.02	2.27 ± 0.01	2.23 ± 0.01
	D32	3.10 ± 0.01**	3.13 ± 0.02***	3.30 ± 0.01***	2.65 ± 0.01	2.70 ± 0.01*	2.67 ± 0.01***
	D48	3.27 ± 0.01**	3.13 ± 0.02***	3.103 ± 0.01***	3.35 ± 0.02***	2.90 ± 0.02**	2.70 ± 0.01***
Protein (g·L^−1^)	WT	0.19 ± 0.01	0.18 ± 0.01	0.19 ± 0.01	0.18 ± 0.01	0.18 ± 0.01	0.18 ± 0.02
	D6	0.34 ± 0.06***	0.28 ± 0.02***	0.28 ± 0.02	0.26 ± 0.02**	0.25 ± 0.02*	0.25 ± 0.02***
	D15	0.35 ± 0.02***	0.35 ± 0.02***	0.40 ± 0.03***	0.14 ± 0.01	0.11 ± 0.00*	0.11 ± 0.01***
	D18	0.35 ± 0.03***	0.40 ± 0.02***	0.38 ± 0.04***	0.32 ± 0.02***	0.21 ± 0.02	0.33 ± 0.01***
	**D24**	0.38 ± 0.03***	0.38 ± 0.02***	0.38 ± 0.04***	0.37 ± 0.03***	0.37 ± 0.05***	0.38 ± 0.02***
	D25	0.25 ± 0.04	0.47 ± 0.04***	0.45 ± 0.07***	0.34 ± 0.03***	0.34 ± 0.02***	0.34 ± 0.01***
	D32	0.27 ± 0.02	0.28 ± 0.02**	0.27 ± 0.01	0.23 ± 0.01	0.22 ± 0.01	0.22 ± 0.00*
	D48	0.39 ± 0.01***	0.44 ± 0.02***	0.19 ± 0.00	0.10 ± 0.01**	0.18 ± 0.03	0.18 ± 0.01

Note: The mycelial biomass and protein yield were measured in cultures without passage (designated as F0) and after 2, 4, 6, 8, and 10 passages (designated as F2, F4, F6, F8, and F10, respectively). Asterisks denote statistically significant differences versus the WT strain (*p < 0.05, **p < 0.01, ***p < 0.001)

It is well established that the antagonistic reaction serves as a convenient test for evaluating somatic incompatibility [80]. Generally, antagonism is considered an incompatible reaction occurring at the hyphal contact zone, manifesting as pigment formation, dense hyphal protrusions, or sparse hyphal growth. When an antagonistic reaction occurs between two fungi, it indicates that they possess distinct genetic characteristics [81]. An antagonistic test conducted between the mutant strain D24 and the parental strain revealed a clear antagonistic reaction, suggesting that mutant strain D24 is genetically different from the WT strain (Fig. S2).

### Comparative analysis of fermentation kinetic characteristics between the original strain and mutant strain D24

**Fig. 7:**
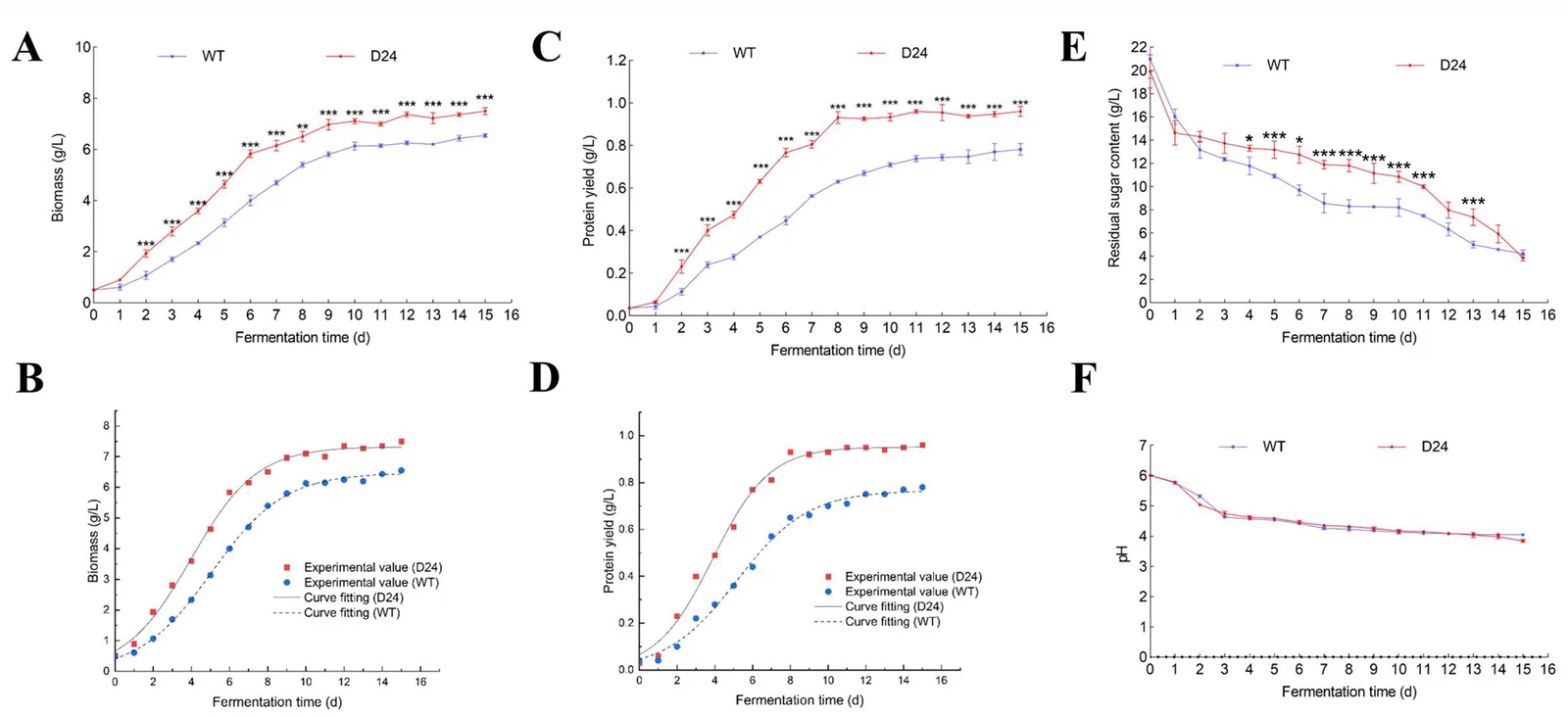
Fermentation kinetic characteristics of *G. frondosa* mutant D24 and the original strain. (**A**) Changes in mycelial biomass during fermentation. (**B**) Fitting curves of mycelial growth kinetics. (**C**) Changes in protein yield during fermentation. (**D**) Fitting curves of protein accumulation kinetics. (**E**) Changes in residual sugar concentration during fermentation. (**F**) Changes in pH during fermentation. Data are the mean ± S.D. from three replicate experiments. *P˂0.05, **P˂0.01, ***P˂0.001 compared with the WT strain

Accumulated evidence reveals that studying fermentation kinetics provides a theoretical basis for optimizing fermentation process control, scaling up laboratory-scale data, and transitioning from batch to fed-batch fermentation. Consequently, this research area has garnered increasing attention from biochemical engineering scholars worldwide [82]. To compare fermentation kinetics between the wild strain and the mutant strain D24, batch cultures were established with periodic monitoring of mycelial biomass, protein production, pH dynamics, and residual sugar concentrations. As depicted in Fig. 7A, with the extension of cultivation time, the biomass of both the WT strain and mutant strain D24 gradually increased before stabilizing. Notably, compared with the WT strain, mutant strain D24 demonstrated significantly enhanced biomass productivity from Day 2 to Day 15 (*p* < 0.01). Moreover, mutant strain D24 displayed a higher growth rate, as evidenced by a steeper slope, indicating stronger environmental adaptability and a shorter lag phase. To further analyze mycelial growth, the data were nonlinearly fitted using Origin 2022. Microbial growth kinetics are commonly modeled using several models, such as the Monod equation or the Logistic equation. Among these models, the Logistic model exhibits a typical S-shaped curve, effectively capturing the inhibitory effect of increasing cell density on microbial growth during batch fermentation. It provides a strong fit for the cell growth dynamics observed in batch fermentation processes [83, 84]. Therefore, the Logistic model was adopted in this study. As shown in Fig. 7B, the Logistic model was effective for fitting the hyphal growth of mutant D24 (*R*^2^ = 0.9965) and the original strains (*R*^2^ = 0.9988). Notably, the mutant D24 demonstrated significantly enhanced fermentation performance compared to the wild-type strain, achieving higher maximum biomass and superior maximum specific growth rate (μmax: 0.5803 ± 0.0256 h^−1^ vs WT 0.5278 ± 0.0138 h^−1^). These findings indicate that the mutated strain exhibited increased mycelium yield and a faster growth rate.

In terms of protein yield, both the wild strain and mutant strain D24 exhibited similar overall trends (Fig. 7C). During the first 7 days and 8 days of fermentation, the proteins of wild and mutant strains accumulated rapidly, respectively. Subsequently, the protein accumulation rate of the two strains clearly slowed down. Moreover, markedly higher protein yield was detected in the mutant strain D24 compared to the original strain after two days of fermentation *(p* < 0.001). Similarly, the Logistic model was also introduced and successfully fitted the protein accumulation results of mutant D24 (*R*^2^ = 0.9922) and the parental strain (*R*^2^ = 0.9947) (Fig. 7D). Moreover, the D24 mutant outperformed the wild-type strain in both protein production and maximum specific protein production rate (μmax improved from 0.5334 ± 0.0306 h^−^^1^ to 0.6669 ± 0.0466 h^−1^). These results provide a basis for the scaled-up production of *G. frondosa* mycelium in subsequent studies.

It is well established that sugar serves as the primary carbon source for microbes during fermentation, playing a crucial role in both microbial growth and product accumulation. Therefore, changes in the residual sugar levels in the fermentation broth were monitored during the fermentation of both strains. As shown in Fig. 7E, residual sugar levels in both groups gradually decreased with increasing cultivation time, which is consistent with previous studies [85]. Given that a certain amount of residual sugar remained at the end of fermentation, it is possible that glucose was not the limiting factor in *G. frondosa* fermentation at this stage. However, at all sampling time points except days 1 and 15, the residual sugar in the mutant strain group was higher than that in the WT strain group, especially from day 4 to day 11 and on day 13. Similar observations have been reported by other researchers. For instance, Picon et al. observed that an *Escherichia coli* mutant strain exhibited a higher protein production ratio than the wild-type strain, along with a lower glucose consumption rate [86]. These results suggest that the *G. frondosa* mutant strain D24 may possess enhanced metabolic efficiency, potentially directing glucose more effectively toward target product biosynthesis and mycelial growth, while possibly reducing carbon flux into competing byproduct pathways.

Additionally, the pH profiles of the two strains were recorded daily during fermentation. The results showed that the pH values of both fermentation broths gradually decreased from an initial value of 6.2 and stabilized at around 4, which aligns with findings from a previous study [85]. Moreover, it can be noted in Fig. 7F that the pH of the mutant strain’s fermentation broth was not significantly different from that of the original strain (*p* > 0.05). According to previous studies, variations in culture pH can markedly alter the synthesis of active substances and overall biomass in some fungi [87, 88]. However, the absence of significant pH differences between the two strains in this study suggests that pH fluctuations were unlikely to be the sole driving factor behind the higher biomass and protein production observed in the mutant strain D24.

### Observation of phenotypic differences between the wild strain and mutant D24 of *G*. *frondosa*

**Fig. 8:**
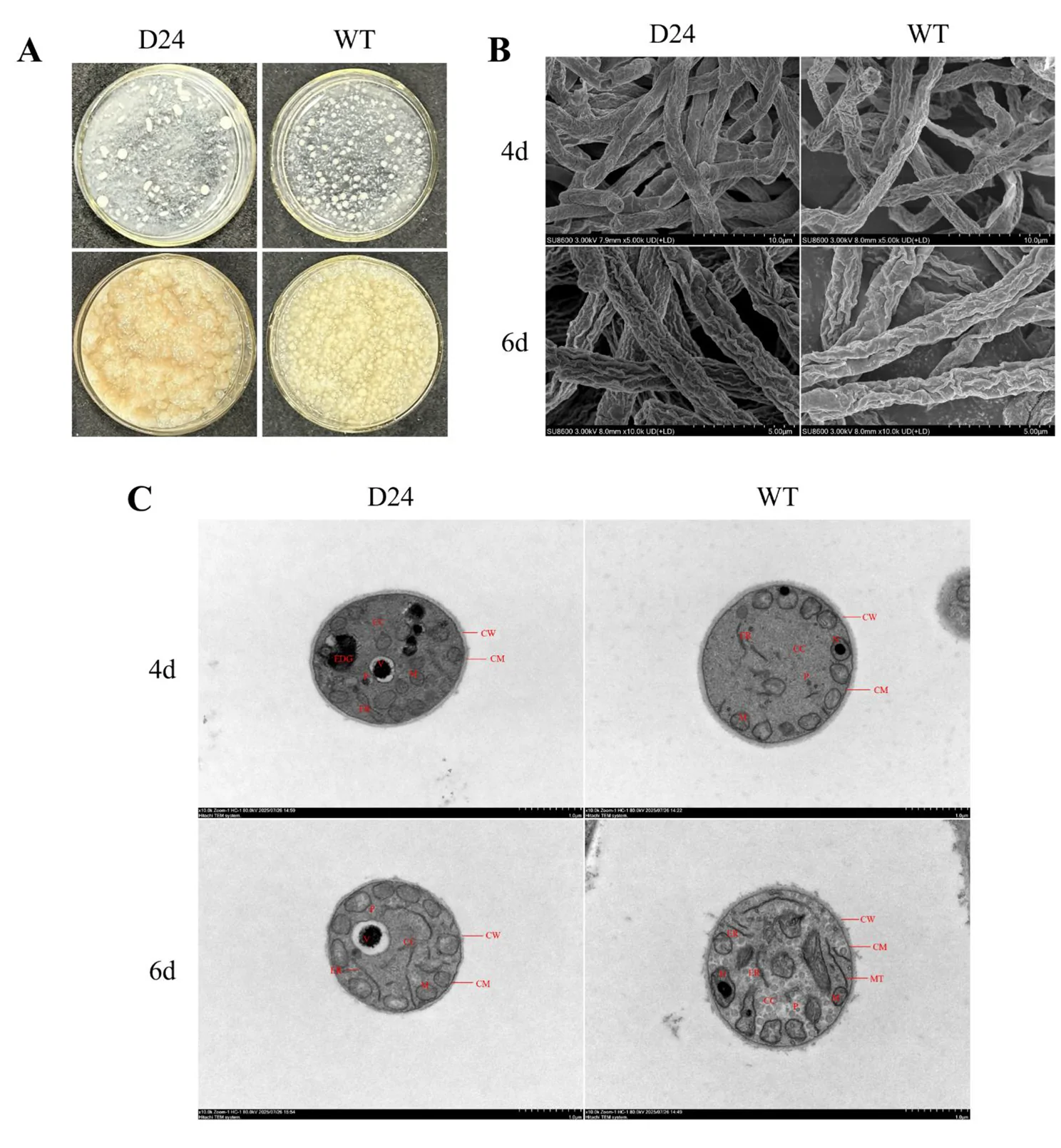
The macroscopic and microscopic morphology of *G. frondosa* D24 and wild strain. (**A**) Representative macroscopic morphology of *G. frondosa* mycelia (WT and D24 strains) after 10 days of liquid culture. Upper panel: mycelial suspension (diluted mycelia suspension using PBS). Lower panel: mycelia harvested after fermentation. (**B**) Representative SEM images of *G. frondosa* D24 and WT strain mycelia cultured for 4 or 6 days. (**C**) Representative TEM images of *G. frondosa* D24 and WT strain mycelia after 4 or 6 days of liquid culture. CW: cell wall, CM: cell membrane, M: mitochondria, ER: endoplasmic reticulum, CC: cytoplasm, P: peroxisome, N: nucleus, EDG: electron dense granules, MT: microtubule, V: vacuole

To assess macroscopic morphological differences, mycelia from the mutant D24 and wild-type strain were collected separately after 10 days of liquid fermentation and photographed. As shown in Fig. 8A, the D24 strain exhibited significantly more flocculent hyphae than the original strain. It is speculated that this morphological trait of the mutant D24 strain likely facilitated oxygen and nutrient transport, thereby enhancing hyphal growth and metabolite synthesis. Previous studies have demonstrated that dense hyphal pellets can restrict substrate and oxygen diffusion, impairing metabolic activity and reducing yield [89]. Since *G. frondosa* is an obligate aerobe, oxygen availability is a key limiting factor for its rapid growth. The flocculent hyphae of D24 promoted greater interfacial contact with oxygen and nutrients, potentially explaining its enhanced biomass accumulation and protein production.

The surface morphology and ultrastructure of two strains grown for 4 days or 6 days were also observed using SEM and TEM, respectively. As indicated in Fig. 8B, similar mycelium pellets formed by entangled hyphae were observed in the samples cultured for 4 or 6 days in both the WT group and the D24 group. Moreover, slight wrinkles were found on the surfaces of the hyphae obtained from the two groups, which was a similar finding to the results provided by others [90]. The majority of the hyphae in the two groups were relatively plump, especially in the D24 group, suggesting vigorous growth of the hyphae in both groups at this stage. At 6 days post-cultivation, SEM images showed that wrinkled and shrunken hyphae were observable in both the D24 and WT groups, which might be suggestive of a growth slowdown under conditions of nutrient depletion and limited space. Additionally, TEM images revealed that after 4 days of cultivation, uniform cell walls (CW), intact cell membranes (CM), uniform cytoplasm (CC), endoplasmic reticulum (ER), and mitochondria (M) were all observed in both strains (Fig. 8C). Furthermore, no obvious cell wall separation was observed in either group, indicating that the hyphal structure of the two strains remains intact. Compared to the WT group, the D24 group exhibited more electron-dense granules (EDG) in the 4-day-old hyphae. Similarly, the growth rate of *Arthroborys flagrans* with knocked out *LaeA* was found to be slowed down, and fewer electron EDGs were also found in it [91]. Nevertheless, the precise biological functions of these EDG remain to be further elucidated in subsequent studies. However, as shown in Fig. 8C, the cell structure of *G. frondosa* hyphae in the WT group at 6 days exhibited significant differences. Compared with WT group hyphae, the D24 exhibited more regular mitochondria and homogeneous cytoplasmic texture, suggesting enhanced metabolic activity of the mutant D24.

### Whole genome resequencing analysis of wild strain and mutant strain of *G*. *frondosa*

#### Statistical analysis and annotation of genomic SNPs and InDels

To investigate the genetic variation of the mutant strain D24, genome resequencing analysis was performed on both the parental strain and the mutant strain D24. After filtering, the clean reads were obtained and aligned with the reference genome, which were summarized in Table S3. It can be clearly noted that the mapping rates for the WT strain and mutant strain D24 were 83.09% and 77.17%, respectively. The proportion of Q30 in each strain sample was demonstrated to be higher than 90%. Moreover, highly genome coverage rates (exceeding 92%) were found in two strains. These data indicate good reference genome coverage and suitability for variant detection analysis.

**Fig. 9:**
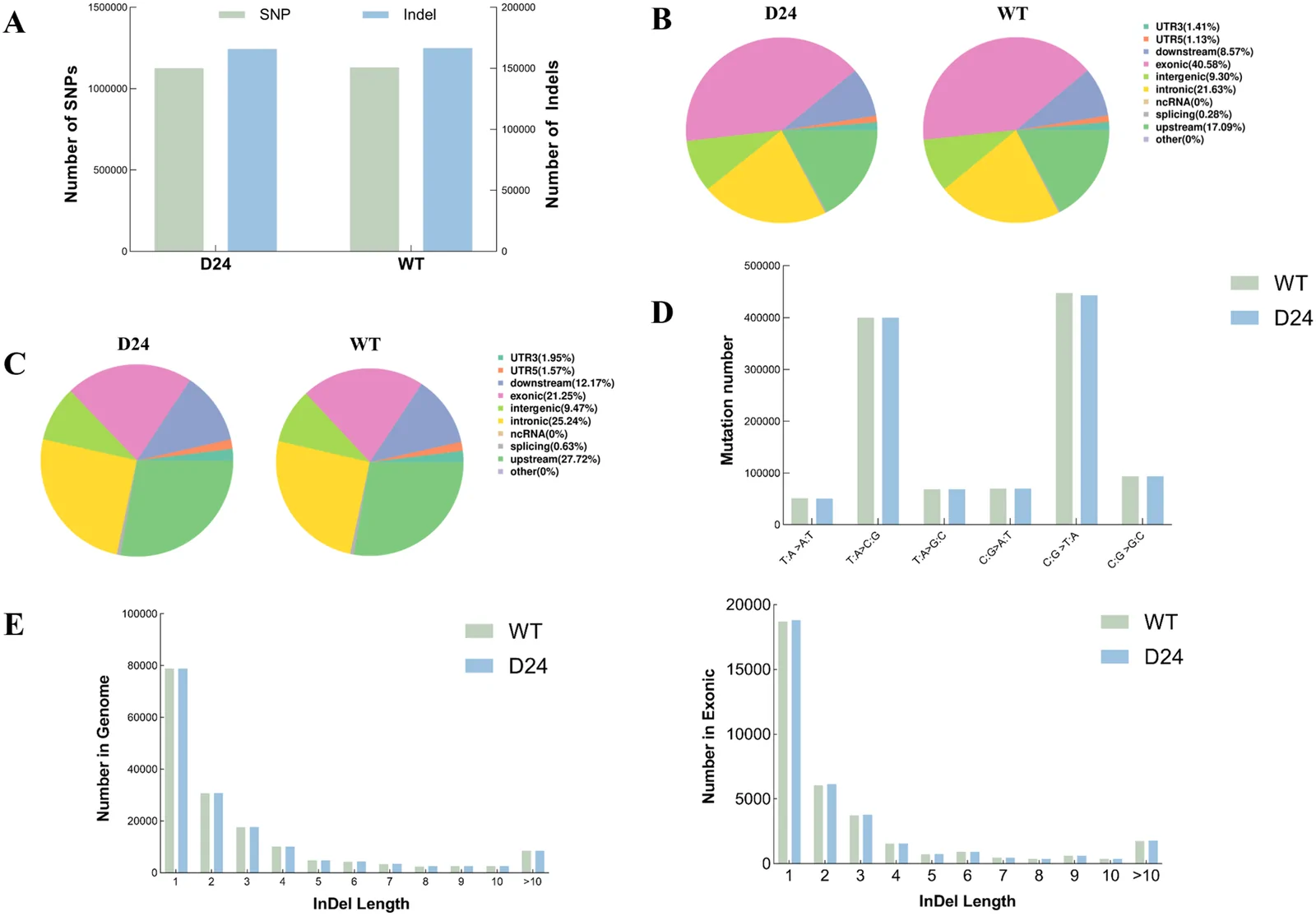
Genome resequencing results of original and selected mutant *G. frondosa* strains. (**A**) Comparative quantification of SNPs and InDels between two strains. (**B**) Distribution of different SNPs. (**C**) Categorization of InDel variants by type. (**D**) Six variation types number of SNPs. (**E**) Length distribution profiles of indels in the whole-genome and exonic region

Furthermore, two common mutational events, namely InDels and SNPs, were introduced to analyze the obtained data. Compared to the reference genome, there were 1,125,718–1,130,586 SNPs and 165,904–166,500 InDels identified in the WT as well as D24 strains, respectively, indicating there were differences between the two strains and the reference genome (Fig. 9A). For the distribution of variant regions, it can be noted that these two strains exhibited a generally similar distribution pattern, and the highest number of SNPs was found to be located in the intronic and exonic regions, while the highest number of InDels was found to be located in the upstream and intronic regions (Figs. 9B and 9C). Additionally, six variation types of SNPs were identified, among which C: G > T: A and T: A > C: G were the most frequent, while T: A > A: T transversions were the least frequent (Fig. 9D). Both strains displayed comparable distributions of SNPs across mutation classes, with the exception of C: G > T: A. It can be seen that, compared with the original strain, the mutant strain D24 had fewer C: G > T: A. Additionally, based on whether they caused amino acid changes, the mutations in the coding region were further classified, including nonsynonymous SNPs, synonymous SNPs, stopgain, stoploss, etc. As illustrated in Table 5, the wild strain had 42 less SNPs with nonsynonymous mutations than the mutant strain D24. There were still a small number of SNPs that acquired or lost stop codons, and the number of SNPs in these two mutation types varied between the tested strains.

**Table 5:** Statistics results of SNPs and InDels annotation

Mutation type		WT	D24
SNPs	Total number of SNPs	1,130,586	1,125,718
	Upstream	95,118	94,665
	Downstream	96,877	96,288
	Synonymous SNP	277953	278166
	Non-synonymous SNP	177050	177092
	Stop gain	3099	3063
	Stop loss	706	700
	Intronic	244,597	243,615
	Exonic	458,808	459,021
	Splicing	3,198	3,237
	Intergenic	105,181	102,123
	UTR5	12,711	12,801
	UTR3	15,906	15,871
	Het_genome	66.23	65.93
InDels	Total number of InDels	165,904	166,500
	Upstream	22,382	22,446
	Downstream	20,197	20,244
	Stopgain	653	664
	Stoploss	136	134
	Frameshift deletion	9,334	9,433
	Frameshift insertion	19,274	19,487
	Nonframeshift deletion	2,998	3,049
	Nonframeshift insertion	2,861	2,903
	Intronic	41,871	41,944
	Splicing	1,037	1,042
	Intergenic	15,717	15,506

Through comparative analysis (Table 5) of the InDels of the two strains, it was found that the mutant strain exhibited more frameshift insertions and frameshift deletions. Similar to the SNPs analysis results, there were differences in the number of InDels that caused stop codon acquisition or loss between the two strains. As illustrated in Fig. 9E, single base insertions or deletions in the genome and exonic region accounted for the vast majority of cases in both strains. Taken together, these findings reveal that, compared with the original strain, the screened mutant strain D24 exhibited different SNPs and InDels, laying the foundation for further exploration of the mechanism of the high-yield performance of the mutant strain D24.

#### Analysis of SNP sites in the mutant strain D24 compared to the original strain

SNP sites of the mutant strain D24 were identified by alignment to the reference genome and then compared with those of the original strain. As shown in Table 6, the transition/transversion (Ti/Tv) ratio of mutant D24 is 2.98 relative to the reference genome and 3.08 relative to the original strain, indicating that DES mutagenesis treatment might tend to induce transition-type base substitutions in *G. frondosa*. A total of 57,216 SNP sites were identified between mutant D24 and the original strain WT, of which 4,068 were non-synonymous. Furthermore, 2,505 genes were found to be involved in these SNPs, and functional annotation analysis of these genes was further performed, including GO functional annotation analysis and KEGG metabolic pathway analysis.

**Table 6:** SNPs statistics of mutant strain D24

Terms	Relative to the reference genome	Relative to the original strain
Total number	1,125,718	57,216
Transition (Ti)	842,862	43,189
Transversion (Tv)	282,856	14,207
The ratio of transition to transversion (Ti/Tv)	2.98	3.08

#### Functional annotation analysis of identified genes

**Fig. 10:**
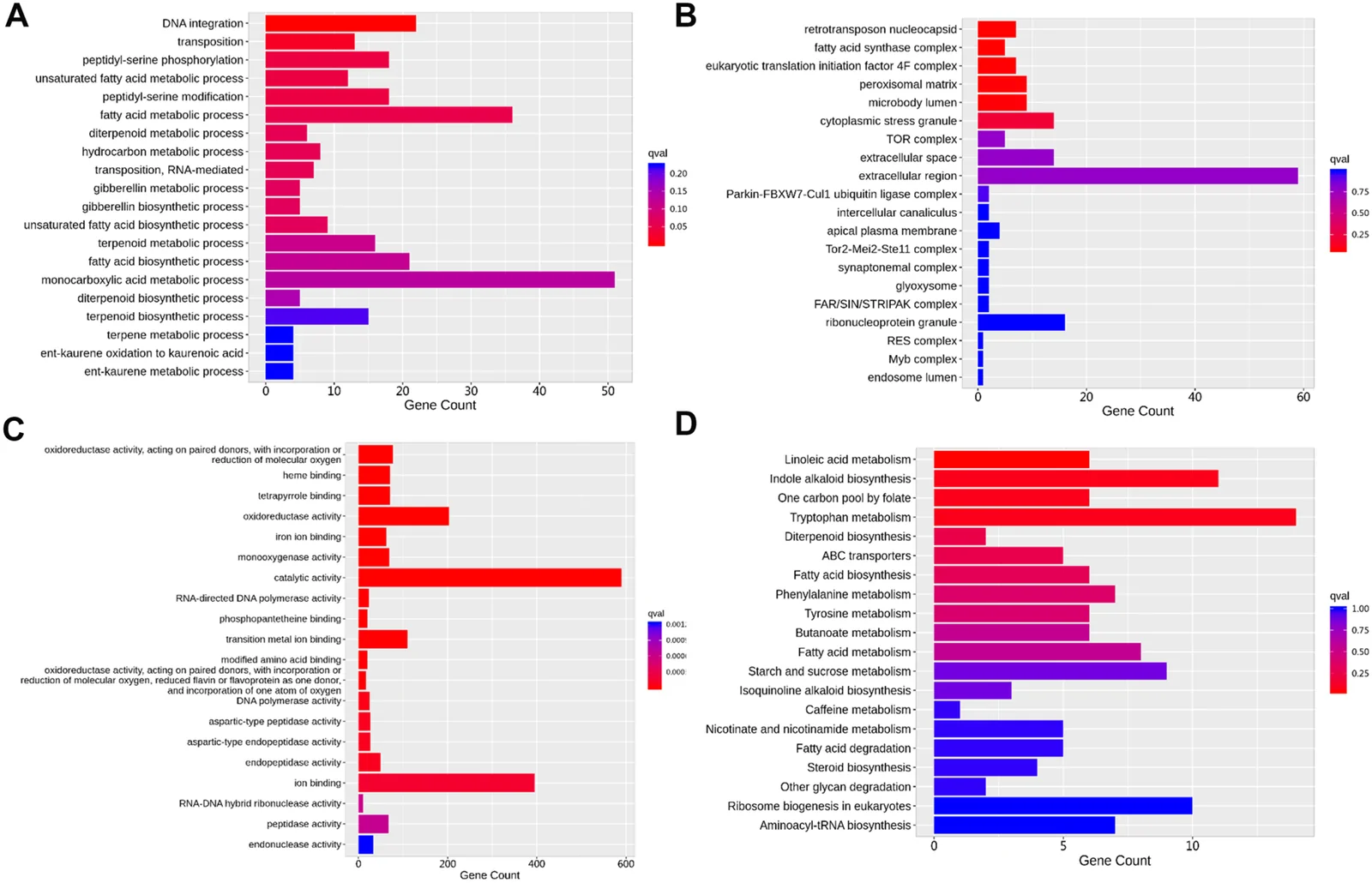
Functional annotation analysis of genes harboring SNPs. (**A-C**) GO functional annotation analysis of genes. (**A**) Biological process; (**B**) Cellular component; (**C**) Molecular function. (**D**) KEGG metabolic pathway analysis of genes

GO functional annotation revealed that the genes harboring SNPs were assigned to three main GO categories: biological process (BP, 2658), cellular component (CC, 476), and molecular function (MF, 1037). For each of the three categories, the top 20 enriched GO terms are presented in Fig. 10. As shown in Fig. 10A, within the BP category, the genes were significantly enriched in transposon-related processes (e.g., DNA integration, transposition, and RNA-mediated transposition); post-translational modification processes of proteins (e.g., peptidyl-serine modification and peptidyl-serine phosphorylation); and metabolic pathways, including unsaturated fatty acid metabolic process, unsaturated fatty acid biosynthetic process, terpenoid metabolic process, terpenoid biosynthetic process, etc. Additionally, these genes were also involved in hydrocarbon metabolic process, monocarboxylic acid metabolic process, and diterpenoid metabolic process. In terms of CC, as shown in Fig. 10B, the genes were associated with retrotransposon nucleocapsid, fatty acid synthase complex, peroxisomal matrix, and cytoplasmic stress granule. As shown in Fig. 10C, the MF category exhibited the highest enrichment significance. The genes were mainly enriched in functional terms such as oxidoreductase activity, RNA-directed DNA polymerase activity, endonuclease activity, and catalytic activity.

Furthermore, KEGG pathway enrichment analysis was performed to elucidate the differences in functional regulation of the mutant strain at the metabolic pathway level (Fig. 10D). The analysis revealed that 34 genes in mutant D24 were involved in amino acid-related pathways (pathways such as tryptophan metabolism, phenylalanine metabolism, tyrosine metabolism, and aminoacyl-tRNA biosynthesis), which may be related to the supply of precursors for protein synthesis. It is well known that amino acids are indispensable nutrients that sustain various life activities. Organisms can utilize these compounds as carbon sources for energy production or as nitrogen sources to support growth and development. Additionally, specific amino acids participate in signal transduction as signaling molecules and serve as protective agents against environmental stress [92]. Additionally, 16 genes are involved in terpenoid and alkaloid metabolism (pathways such as indole alkaloid biosynthesis, diterpenoid biosynthesis, and isoquinoline alkaloid biosynthesis), suggesting that secondary metabolic pathways might be altered. In addition, some genes are also involved in carbohydrate metabolism and ABC transporters. Furthermore, 29 genes are involved in lipid metabolism (pathways such as linoleic acid metabolism, fatty acid biosynthesis, fatty acid metabolism, steroid biosynthesis, and fatty acid degradation), which may affect cell membrane composition and energy metabolism. Of particular note, the linoleic acid metabolism pathway was the most significantly enriched one. The linoleic acid metabolism pathway is a core metabolic network in living organisms that converts the essential fatty acid linoleic acid into a range of metabolites, including oxylipins. Oxylipins are oxygenated lipid species generated from polyunsaturated fatty acids through either enzymatic or non-enzymatic oxygenation reactions, including hydroxyoctadecaenoic acid (HODE), hydroperoxyoctadecadienoic acid (HPODE), etc. [93]. Previous studies have revealed that oxylipins exhibit crucial biological activities, functioning as signaling factors for communication both within and between cells and as regulators of microbial metabolism [94, 95]. In present work, two genes were enriched in this pathway, including *PpoC* and *CYP3A*-like gene. Among them, *PpoC* encodes linoleate 10 R-lipoxygenase, a LOX (lipoxygenases) enzyme that catalyzes the oxidation of linoleic acid to produce hydroperoxyoctadecadienoic acid (HPODE) [96]. This intermediate can then be further metabolized by other enzymes into oxylipins (hydroxyoctadecadienoic acid (HODE) and di-hydroxyoctadecadienoic acid (diHODE)) [97]. Meanwhile, it was reported that deletion of the *PpoC* allele also reduced spore development in *Aspergillus nidulans* [96]. Additionally, a gene encoding a CYP3A-like cytochrome P450 (CYP450) was identified in this pathway. In fungi, linoleic acid can be oxidized by linoleate 8-dioxygenase (8-DOX) into hydroperoxides, which are then converted by the CYP450 enzymes into diols, compounds that promote hyphal branching and development in filamentous fungi [97]. Taken together, it is speculated that genetic variations in *CYP3A*-like and *PpoC* may influence the linoleic acid metabolism, leading to increased production of oxylipins, including diols, thereby promoting hyphal growth of *G. frondosa*. Although the absolute yield of D24 requires further improvement before direct industrial application, the candidate mutations identified here offer valuable targets for future strain engineering, whether through introduction into superior backgrounds or via application of the same mutagenesis pipeline to other strains.

## Conclusions

In this study, a high-efficiency protoplast preparation and DES mutagenesis system was established for *G. frondosa*, yielding a genetically stable mutant strain, D24, with significantly improved mycelial biomass and protein yield. Phenotypic and fermentation kinetic analyses revealed that D24 exhibits accelerated growth kinetics, altered hyphal morphology, and enhanced mitochondrial abundance, which may indicate a shift toward more active metabolic and biosynthetic activity. Additionally, genome resequencing identified 57,216 SNPs in the D24 strain compared with the WT strain, including 4,068 non-synonymous mutations involving 2,505 genes. GO and KEGG enrichment analyses further revealed that these genes were potentially involved in pathways related to amino acid metabolism, lipid metabolism, terpenoid and alkaloid synthesis, and other biological processes. Overall, this study provides a novel high-yielding mutant strain of *G. frondosa*, offering promising material for the development of *G. frondosa*. Nevertheless, further research is still needed to elucidate the underlying mechanisms responsible for this action of the mutant strain. Additionally, the biofunctional properties of this novel strain require additional characterization.

## Electronic supplementary material

Below is the link to the electronic supplementary material.

Supplementary Material 1

## Data Availability

All data supporting the findings of this study are available within the paper and its Supplementary Information.
