# Supplementary material for: Process optimization for protoplast preparation from Grifola frondosa and development of a high-yield mutant with enhanced mycelial growth and protein production
Source: BMC Biotechnol. 2026 Jul 8;26:116. doi: 10.1186/s12896-026-01197-z (PMC13628877; doi:10.1186/s12896-026-01197-z)
Supplement: Supplementary file 1 [file 12896_2026_1197_MOESM1_ESM.docx]

**Process optimization for protoplast preparation from *Grifola frondosa* and development of a high-yield mutant with enhanced mycelial growth and protein production**

**Qiaoyu Wang†, Ziteng Zhao†, Qiuyu Lu, Ke'er Xiao, Lingyu Gao, Mukaddas Sai, Lili Guan*, Linna Du***

Engineering Research Center of Bioreactor and Pharmaceutical Development, Ministry of Education, College of Life Science, Jilin Agricultural University, No. 2888, Xincheng Street, Changchun 130118, China. [13634320796@126.com](mailto:13634320796@126.com) (Q.Y.W.); [Zhaoziteng2002@163.com(Z.T.Z)](mailto:Zhaoziteng2002@163.com(Z.T.Z)); 13278503717@163.com(Q.Y.L); [18943912157@163.com](mailto:18943912157@163.com) (K.E.X.); 17682371741@163.com (M.K.S); [13153131973@163.com(L.Y.G)](mailto:13153131973@163.com(L.Y.G))

*****	Correspondence: dulinna0918@163.com; [llguan@jlau.edu.cn (L.L.G)](mailto:llguan@jlau.edu.cn(L.L.G))

**^†^**	These authors contributed equally to this work.

*** Correspondence:**

Linna Du, Ph.D

Engineering Research Center of Bioreactor and Pharmaceutical Development, Ministry of Education, College of Life Science, Jilin Agricultural University, Changchun, China

Email: [dulinna0918@163.com](mailto:dulinna0918@163.com;)

Lili Guan, Ph.D

Engineering Research Center of Bioreactor and Pharmaceutical Development, Ministry of Education, College of Life Science, Jilin Agricultural University, Changchun, China

Email: [llguan@jlau.edu.cn](mailto:llguan@jlau.edu.cn(L.L.G))

**Table S1**. Analysis of variance for model obtained in Plackett-Burman experiment

| Source | Degree of freedom | Adj Sum of squares | Adj Mean square | *F*-value | *P*-value |
| --- | --- | --- | --- | --- | --- |
| Model | 8 | 13.2799 | 1.65998 | 55.86 | 0.004 |
| Linear | 8 | 13.2799 | 1.65998 | 55.86 | 0.004 |
| *X*_1_ | 1 | 5.9221 | 5.92207 | 199.27 | 0.001 |
| *X*_2_ | 1 | 0.8060 | 0.80601 | 27.12 | 0.014 |
| *X*_3_ | 1 | 3.7074 | 3.70741 | 124.75 | 0.002 |
| *X*_4_ | 1 | 2.6791 | 2.67907 | 90.15 | 0.002 |
| *X*_5_ | 1 | 0.1180 | 0.11801 | 3.97 | 0.140 |
| *X*_6_ | 1 | 0.0024 | 0.00241 | 0.08 | 0.794 |
| *X*_7_ | 1 | 0.0052 | 0.00521 | 0.18 | 0.704 |
| *X*_8_ | 1 | 0.0397 | 0.03968 | 1.33 | 0.332 |
| Pure error | 3 | 0.0892 | 0.02972 |  |  |
| Total | 11 | 13.3690 |  |  |  |

**Table S2.** Efficiency comparison of ANN-GA and RSM in optimizing the protoplast preparation process of *G. frondosa*

| Parameters | Protoplast yield (10^6^ protoplasts/g FW) | |
| --- | --- | --- |
|  | RSM | ANN |
| *R*^2^ | 0.966 | 0.954 |
| S.D., % | 5.626 | 5.824 |
| MSE | 0.051 | 0.068 |
| RMSE | 0.226 | 0.260 |
| χ^2^ | 0.347 | 0.494 |
| SEP, % | 0.052 | 0.060 |

**Table S3.** Statistics of the alignment with the reference genome

| Samples | D24 | WT |
| --- | --- | --- |
| Genome Length | 39,289,314 | 39,289,314 |
| Clean Reads | 54,060,482 | 27,769,308 |
| Clean Bases | 8,109,072,300 | 4,165,396,200 |
| Mapped Bases | 6,258,167,745 | 3,461,153,947 |
| Mapping Rate (%) | 77.17 | 83.09 |
| Duplication Rate (%) | 29.33 | 24.58 |
| Uniq Rate (%) | 86.26 | 88.79 |
| Mean Depth | 171.01 | 92.47 |
| Raw Reads(M) | 54.06 | 27.76 |
| Raw Base(G) | 8.11 | 4.17 |
| Q30(%) | 90.2 | 92.6 |
| Coverage Rate (%) (≥1X) | 93.03 | 92.67 |

**Figure S1.** Pareto chart for the standardized influence of the test variables on the yield of protoplasts

**Figure S2.** Antagonism assay of *G. frondosa* D24 versus wild-type strain (WT)
